# Diagnostic and prognostic value of circulating biomarkers in heart failure

**DOI:** 10.3389/fcvm.2025.1633164

**Published:** 2025-09-24

**Authors:** Artur Kovenskiy, Zhussipbek Mukhatayev, Aliya Sailybayeva, Makhabbat Bekbossynova, Almagul Kushugulova

**Affiliations:** ^1^ National Laboratory Astana, Nazarbayev University, Astana, Kazakhstan; ^2^ CF “University Medical Center”, Heart Center, Astana, Kazakhstan

**Keywords:** heart failure, biomarker, meta-analysis, inflammation, comorbidities

## Abstract

**Background:**

Heart failure (HF) represents a global health burden with distinct phenotypes characterized by varying left ventricular ejection fraction (LVEF). Despite shared endothelial dysfunction, heart failure with reduced (HFrEF) and preserved ejection fraction (HFpEF) exhibit fundamentally different pathophysiological mechanisms, comorbidity profiles, and treatment responses.

**Methods:**

This systematic review and meta-analysis examine inflammatory, cardiac remodelling and congestion, and myocardial injury biomarkers across HF phenotypes, integrating data from 78 studies encompassing 58,076 subjects.

**Results:**

Our analysis reveals a significant elevation of IL-6, TNF-alpha, and hs-CRP in HF compared to controls, with distinct biomarker profiles emerging between phenotypes. While inflammatory markers universally increase with disease severity, their utility in phenotypic differentiation remains limited due to substantial overlap. Comorbidity burden significantly influences inflammatory profiles, creating diagnostic challenges that multi-biomarker approaches may address. NT-proBNP, sST2, GDF-15, and cardiac troponins demonstrate complementary value when combined with inflammatory markers, potentially enabling more precise phenotypic classification.

**Conclusion:**

Our findings highlight the central role of inflammation in HF pathophysiology while identifying critical knowledge gaps, particularly regarding HFpEF-specific inflammatory signatures. This comprehensive analysis provides a foundation for developing targeted immunomodulatory therapies and personalized diagnostic approaches in heart failure management.

**Systematic Review Registration:**

https://www.crd.york.ac.uk/PROSPERO/view/CRD42025639405, PROSPERO CRD42025639405.

## 1 Introduction

Heart failure (HF) represents a global health crisis, significantly contributing to mortality, disability, and morbidity worldwide (1). Recent advances in our understanding of HF have revealed its complex nature, with three distinct phenotypes based on Left Ventricular Ejection Fraction (LVEF): Heart Failure with Reduced Ejection Fraction (HFrEF, LVEF ≤40%), Heart Failure with Preserved Ejection Fraction (HFpEF, LVEF ≥50%), and the intermediary Heart Failure with mildly reduced Ejection Fraction (HFmrEF, LVEF 41%–49%) (2). This phenotypic variation is crucial, as it reflects differences in etiology, demographics, comorbidities, and therapeutic responses (3–5). While HFrEF and HFpEF share risk factors and comorbidities (6), they exhibit distinct gender predispositions and underlying mechanisms. HFpEF often results from chronic inflammation associated with conditions like obesity and diabetes, leading to microvascular dysfunction and oxidative stress (7). In contrast, HFrEF stems from various etiologies including ischemic cardiomyopathy, arrhythmogenic factors, and direct cardiac insults (7–9). Regardless of the distinct phenotypic differences of HFmrEF, its treatment strategy usually considered alike with that of HFpEF (10).

The use of biomarkers in diagnosis and management of HF patients under thoroughly investigation. Among them NT-proBNP is the most promising biomarker, which is currently the most used one aiding to make diagnosis and prognosis of HF development (11). However, debates arise across usage of NT-proBNP and other biomarkers in HF, as individually they are not specific for HF pathogenesis (12). Therefore, there is need in construction of multibiomarkeral approach, targeting novel therapeutical strategy to diagnosis and management of CHF and its distinct phenotypes (13).

**Figure 1:**
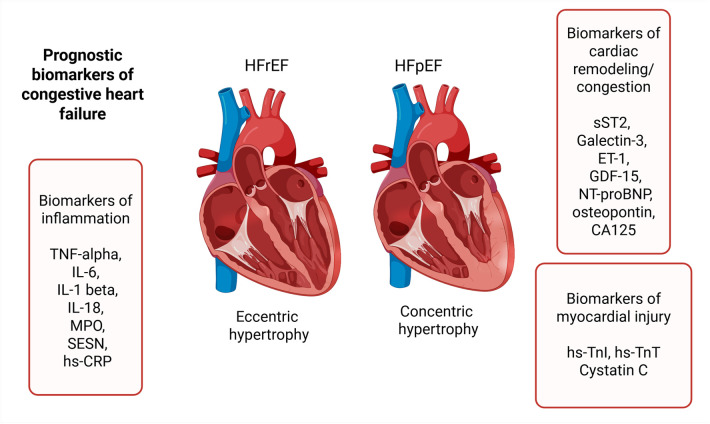
Prognostic biomarkers of heart failure. TNF-alpha, tumor necrosis factor alpha; IL-6, interleukin-6; IL-1 beta, interleukin-1 beta; IL-18, interleukin-18; MPO, myeloperoxidase; SESN, sestrin proteins; hs-CRP, high-sensitivity C-reactive protein; sST2, soluble suppression of tumorigenesis-2; ET-1, endothelin-1; GDF-15, growth differentiation factor-15; NT-proBNP, N-terminal prohormone of brain natriuretic peptide; hs-TnI, high-sensitivity troponin I; hs-TnT, high-sensitivity troponin T.

Biomarkers describing HF have no strict classification, and individually can fall into more than one category. To address this complexity and to facilitate a clearer comprehension, we have categorized them based on their primary association with pathogenetic processes, namely: biomarkers of inflammation, biomarkers of cardiac remodeling and congestion, and biomarkers of myocardial injury (Figure 1).

This systematic review is going to emphasize diagnostic capability of HF biomarkers individually and from multibiomarkeral perspective. This review synthesizes current understanding of biomarkers in HF, highlighting their potential as diagnostic tools and therapeutic targets. By elucidating the complex interplay between inflammation, cardiac remodeling, congestion, myocardial injury and heart failure, we pave the way for personalized medicine approaches in HF management, potentially revolutionizing patient care and outcomes in this devastating disease.

## 2 Methods

**Figure 2:**
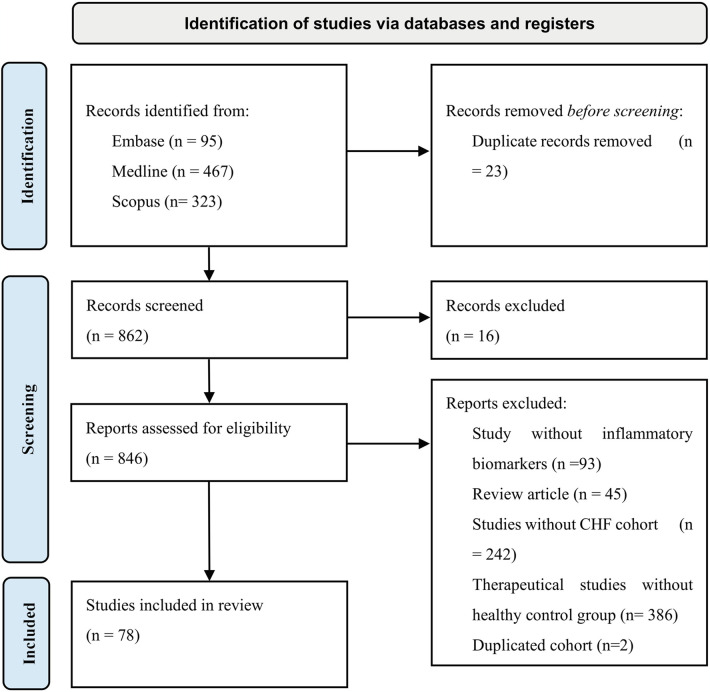
PRISMA flowchart of study inclusion and exclusion criteria.

Medline, Scopus and Embase databases were used in order to collect all eligible data for our study. Search strategy included our study population (using both terms “congestive heart failure” and “heart failure” to capture all relevant publications) and biomarkers with previous evidence (hs-CRP, TNF-alpha, soluble TNF receptors, IL-1, IL-6, IL-18, MPO, Sestrin proteins, sST2, GDF-15, Galectin-3, ET-1, NT-proBNP, osteopontin, cTnT, cTnI, Cystatin C). In addition, only English and human subject type articles were included (Supplementary Table S1). CRP is secreted during most of the inflammation responses that can be triggered by infection, tissue damage (14). Because CRP secretion is not specific and gives poor clinical information, articles that included only non-specific CRP were excluded. Inclusion criteria was having high-sensitivity C-reactive protein (hsCRP), which was recognized as inflammation marker that predicts reverse cardiovascular events (15). Search was conducted on 27 December 2024 and managed by EndNote. Following PRISMA guideline inclusion and exclusion criteria was conducted and illustrated in Figure 2 (16). 885 articles were collected using Medline, Scopus and Embase databases. Twenty-three duplicates were removed using EndNote (*n* = 23), after those 16 articles were excluded during abstracts screening, 13 were duplicates, two written not in English language and one was animal study. Full text assessment for eligibility excluded 768 studies due to various reasons including studies lacking inflammatory biomarkers from our search strategy (*n* = 93), review articles (*n* = 45), studies not included congestive heart failure patients (*n* = 242), therapeutical studies without healthy control group were also excluded due to inability to assess diagnostic role of biomarkers following drag intervention (*n* = 386), duplicated cohort (*n* = 2). 78 studies were included after screening and assessment for eligibility. The protocol was registered in PROSPERO (CRD42025639405).

Risk of bias assessment was performed for all included studies by risk of bias tool (RoB 2). Risk of bias graph and summary were designed by Review Manager 5 software (Supplementary Figures S1, S2).

The following study characteristics were extracted independently by two reviewers into Microsoft Excel 2021 (version 2108): First author, publication year, title, study design, number of subjects, age, HF type, comorbidities, inflammation biomarkers, evaluated results. Subgroup meta-analysis was done by Review Manager 5 (version 5.4) software to compare sST2, GDF-15, NT-proBNP, IL-6, hs-TnT, hs-TnI concentrations between HF phenotypes and IL-6, TNF-alpha, hs-CRP concentrations between HF and healthy control cohorts. Meta-analysis was performed using standard deviation mean difference (IV, Random effects, 95% CI). Studies reporting concentrations with interquartile ranges were recalculated using method of Wan et al. (17).

## 3 Results

A total of 58,076 subjects from 78 studies were included in this systematic review, with a mean patient age of over 65 years. Study characteristics of included studies are presented in Supplementary Tables S2–S4.

### 3.1 Inflammatory biomarkers

Only seven studies included HF patients with preserved ejection fraction. The remaining studies included only HFrEF patients or had a mixed cohort with predominantly HFrEF patients compared to HFpEF. Only few articles studied IL-1 beta, showing IL-1beta concentration being lower than detection limit in Almasood's study and significantly elevated in congestive heart failure (CHF) cohort compared to control group in Stanciu's study (18, 19). Despite the theoretical basis suggesting that IL-18 and sestrin proteins play a role in the inflammation associated with heart failure (20–22), the search did not find articles measuring IL-18 and sestrin protein concentrations.

#### 3.1.1 IL-6

Abernethy found that IL-6 levels were higher in the acute decompensated heart failure with preserved ejection fraction (AD-HFpEF) compared to stable HFpEF (S-HFpEF) (23). Pandhi found that IL-6 was significantly elevated in patients with severe congestion HF (24). Almasood's study showed that IL-6 levels correlates with NYHA class, indicating severity. Aulin suggested that IL-6, along with other biomarkers, could improve the identification of the risk of developing or worsening HF (25). However, Niebauer's study found no association between IL-6 and CHF worsening (26). Based on a median follow-up duration of 1.9 years, IL-6 demonstrated a stronger association with mortality in the healthy control (HC) group compared to the HF groups (25). Susa reported that IL-6 level do not change between chronic HF patients with and without cardiac events (27), but Davarzani's results of 19-month follow-up of congestive HF patients concluded that the event group had higher IL-6 levels compared to no-event group (28). Boulogne reported that IL-6, among other biomarkers, showed no difference between acute and chronic HF cohorts (29).

**Figure 3:**
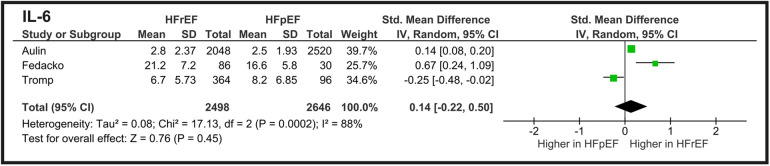
Meta-analysis of IL-6 concentration comparing HF phenotypes.

Meta-analysis comparing IL-6 concentration (ng/L) between HFrEF and HFpEF groups included three studies, as illustrated in Figure 3. The total SMD was 0.14 (95% CI: −0.22 to 0.50) with *p* = 0.45, indicating no statistically significant difference in IL-6 levels between HFrEF and HFpEF. The heterogeneity of the analysis is high (*I*^2^ = 88%), showing that the results vary substantially across studies.

#### 3.1.2 TNF-alpha

Tromp reported that TNF-alpha levels don't differ significantly between HFrEF and HFpEF phenotypes (30). According to Almasood's and Nakamura's work, TNF-alpha levels correlated with NYHA class severity (18, 31). Fedacko indicated that TNF-alpha can be related to CHF cause and severity, while Richter showed that TNF-alpha can predict all-cause mortality in the HF population (32, 33). On the other hand, Susa and Niebauer reported that TNF-alpha is not associated with adverse outcomes in HF (26, 27).

#### 3.1.3 hs-CRP

Cakmak found a positive correlation between novel HF biomarkers, microRNAs, and hs-CRP levels (34). Dubrock reported that a high hs-CRP levels were associated with a greater comorbidity burden, younger age, higher NT-proBNP levels, right ventricular dysfunction, reduced exercise tolerance and chronic obstructive pulmonary disease (COPD) (35). However, 40% of HFpEF patients had hs-CRP levels within the normal range, and no correlation with NYHA functional class was observed (35).

### 3.2 Cardiac remodeling and congestion biomarkers

Andersson revealed that during heart failure, endothelin A receptor mediated vasodilation is primarily diminished, despite the fact that endothelin-1 level is high (36). Pandhi reported that congestion elevates endothelin-1 levels (24). Mohebi's study indicates that endothelin-1 can be considered as reliable predictor of adverse outcomes in HF (37), while Galindo-Fraga reported that endothelin is associated with poor prognosis in HF severity (38).

According to Tromp and Boulogne, Galectin-3 levels weren't significantly different between phenotypes or between the acute and chronic forms of HF (29, 30). Mohebi reported that Galectin-3 is a significant predictor of hospitalization and cardiovascular death (37). Gocer proposed Galectin-3 concentration ranges according to HF severity: “100–460 pg/ml” for mild, “460–1,170 pg/ml” for moderate and “>1,170 pg/ml” for severe HF (39). Galectin-3 levels were significantly correlated with diabetes mellitus, but not with COPD (40, 41).

There is a limited number of studies with measuring osteopontin levels. According to Tromp's study, its levels do not differ between phenotypic groups (30). Osteopontin is a good predictor of adverse outcomes. According to Behnes, its predicting value is higher compared to NT-proBNP (42).

#### 3.2.1 sST2

According to Mohebi, sST2 is a reliable biomarker for predicting adverse outcomes in advanced HF patients (37). The same conclusion was made by Davarzani and Bahuleyan, who found association of sST2 with adverse outcomes and cardiac events (28, 43). However, Boulogne reported opposite results, additionally indicating that sST2 levels do not differ between the acute and chronic forms of HF (29). Crnko made an important observation, indicating that sST2 levels fluctuate during the day, with the highest concentration in the afternoon, and the lowest at night (44). This finding suggests considering blood sample collection time to improve the prognostic potential of sST2. Menghoum's study showed a significant elevation of sST2 in HFpEF compared to control subjects (45). On the contrary, Firouzabadi reported no significant difference in sST2 concentrations between HF and control groups (46). According to the included studies, sST2 does not correlate with the comorbid conditions of diabetes mellitus and COPD, but it showed significance in predicting HF patients with cachexia (40, 41, 47).

**Figure 4:**
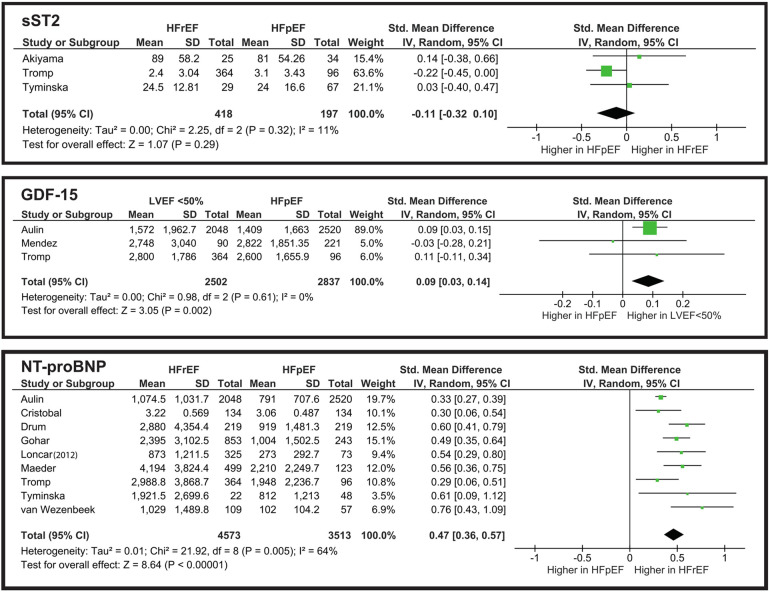
Meta-analysis of cardiac remodeling and congestion biomarkers comparing HF phenotypes.

From the meta-analysis illustrated in Figure 4, the overall SMD is −0.11, indicating that sST2 levels are slightly higher in the HFpEF group. However, the still do not show a significant difference. The overall effect is *Z* = 1.07 (*P* = 0.29), and all three studies cross the line of no effect. Heterogeneity is low (*I*^2^ = 11%), showing that the studies are consistent and do not vary drastically.

#### 3.2.2 GDF-15

Mendez-Fernandez observed that GDF-15 is an independent predictor of all-cause mortality in HF patients with LVEF “>40%” (48). Similarly, Teramoto reported that GDF-15 predicts cardiovascular endpoints, but specifically in elderly patients (49). Davarzani's study shows that GDF-15 is associated with cardiac events in CHF patients (28). When comparing CHF with comorbidities, Ehteshami-Afshar reported significant elevation of GDF-15 in CHF patients with COPD (41).

From the meta-analysis illustrated in Figure 4, we evaluated that while it reaches statistical significance *Z* = 3.05 (*P* = 0.002), its clinical implementation for distinguishing HFpEF from LVEF “<50%” is poor. The heterogeneity is significantly low (*I*^2^ = 0%), which may suggest the need for further studies to establish whether GDF-15 truly aids in phenotype differentiation in clinical practice.

#### 3.2.3 NT-proBNP

The meta-analysis illustrated in Figure 4 shows that NT-proBNP has a statistically significant difference when comparing HFrEF and HFpEF groups. However, in clinical terms this difference is limited. The SMD of 0.47 corresponds to a small-to-moderate effect size. Nonetheless, in combination with other biomarkers NT-proBNP can be a valid diagnostic tool.

### 3.3 Biomarkers of myocardial injury

#### 3.3.1 hs-TnT

**Figure 5:**
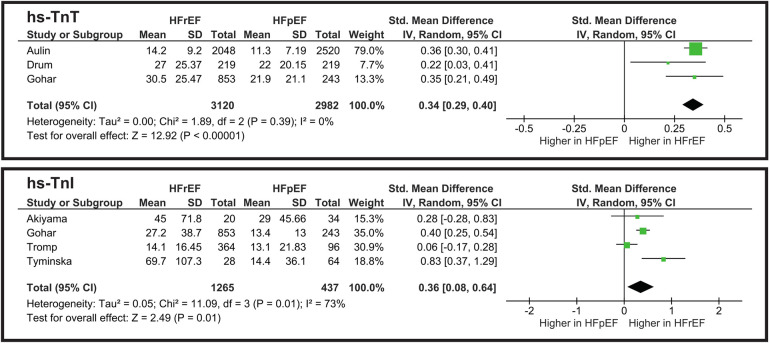
Meta-analysis of myocardial injury biomarkers comparing HF phenotypes.

The meta-analysis illustrated in Figure 5 shows a statistically significant difference between HFpEF and HFrEF. However, an SMD of 0.34 indicates that hs-TnT levels highly overlap between the two groups, thus limiting its clinical utility for phenotype differentiation. Heterogeneity is low (*I*^2^ = 0%), showing the consistency of the studies.

#### 3.3.2 hs-TnI

The pooled standardized mean difference (SMD) for hs-TnI is approximately 0.36 (*p* = 0.01), favoring slightly higher levels in HFpEF (Figure 5). This is a small-to-moderate effect size, suggesting a partial overlap between the two HF subtypes. Heterogeneity is *I*^2^ = 73%, indicating moderate variability among studies. While hs-TnI levels differ statistically between HFrEF and HFpEF, the difference is not large enough to serve as a strong phenotypic discriminator on its own.

#### 3.3.3 Cystatin C

According to Mohebi's study Cystatin C levels significantly increase with HF severity (37). Aulin reported that cystatin C is associated with HF hospitalization and death (25). Both Aulin and Akiyama indicated that Cystatin C does not show a significant difference between phenotypic groups and cannot be used to clinically differentiate them (25, 50).

## 4 Discussion

This systematic review represents the most up-to-date analysis on biomarkers related to HF. Everett's study showed a promising future for anti-cytokine treatments, a monoclonal antibody against IL-1 beta significantly decreased hospitalization and mortality, which reveals importance of chronic inflammation in CHF pathogenesis (51). Additionally, the concentration of inflammatory biomarkers is positively correlated with the number of comorbid conditions, complicating the differentiation between HFpEF and HFrEF (35). Aulin's research demonstrated the biomechanical stress prevalence in HFrEF, showing higher NT-proBNP levels in HFrEF (1,074 ng/L) compared to HfpEF (791 ng/L) (25). The plasma NT-proBNP threshold for inclusion criteria in heart failure patients varies greatly. In Pandhi's study, the threshold was NT-proBNP “>2,000 pg/ml”, whereas in Dubrock's study, it was NT-proBNP “>400 pg/ml” (24, 35). In Stanciu's study both coronary sinus (CS) and peripheral venous (PV) NT-proBNP concentration correlated to CS IL-6, IL1-beta and TNF-alpha levels (19). According to the results of the CORONA clinical trials, NT-proBNP was the strongest predictor of death from worsening heart failure during the 3-month follow-up period (52, 53).

Normal biomarker values, according to Boulogne's and Dubrock's research are the following: hs-CRP “<3 mg/L”, TNF-alpha “<6 pg/ml”, IL-6 “<7 pg/ml”, MPO “<50 pg/ml”, ST2 “<35 ng/ml”, GDF-15 “<1,200 ng/L”, Gal-3 “<10 ng/ml” (29, 35). The threshold levels of biomarkers for detecting cachectic HF are “>5 mg/L” for hs-CRP and “>4 pg/ml” for IL-6 (54). According to Nakamura's study, the normal value for hs-CRP was 0.02 mg/dl, and for TNF-alpha, it was 3.8 pg/ml (31). In Everett's study, patients with hs-CRP levels “<2 mg/L” were considered to have achieved treatment success, indicating the treatment threshold as established in the CANTOS clinical trial (51). Thibodeau reported elevated threshold levels of NT-proBNP and hs-TnT as 1,000 pg/ml and 52 ng/L, respectively (55). NT-proBNP level standards are age-dependent, and increase with older age (56). Chenevier-Gobeaux reported NT-proBNP threshold values as 1,700 pg/ml for patients “<85 years old” and 2,800 pg/ml for those “>85 years old” with CHF. Maeder's treatment strategy focused on reducing NT-proBNP below the inclusion criteria: “<400 ng/L” in patients “<75 years old”, “<800 ng/L” in those “≥75 years old” (57). According to the literature, NT-proBNP-guided therapy improves disease management and shows a trend toward cost reduction, with the highest cost-effectiveness in HF patients aged 60–75 years with two or fewer comorbidities (58, 59).

While some biomarkers, such as NT-proBNP and hs-TnT, remain central to diagnosis and prognosis, others, including ET-1, sST2, and GDF-15, show potential but require further validation. Multi-biomarker approaches may enhance predictive accuracy, though clinical implementation remains challenging due to biomarker overlap and variability. The multi-biomarker approach has demonstrated superior predictive value compared to single-biomarker assessments in heart failure prognosis. Richter reported that a combination of NT-proBNP, hs-TnT, TIMP-1, GDF-15, and IBP-4 provided more accurate predictions than relying solely on NT-proBNP (33). Similarly, Wright observed that combining NT-proBNP with urocortin levels enhanced the ability to predict heart failure outcomes more effectively than using either marker alone (60). Lupón's findings further support this approach, indicating that hs-cTnT and hs-ST2 together offer better prognostic accuracy than when combined with NT-proBNP (61). The meta-analysis illustrated in Figure 4 underscores that while NT-proBNP alone shows a statistically significant difference between HFrEF and HFpEF, its clinical utility is limited. However, when integrated into a multimarker panel, NT-proBNP significantly enhances the diagnostic and prognostic capabilities, emphasizing the potential of a comprehensive biomarker strategy in heart failure management. Standardizing sampling protocols, particularly for time-sensitive biomarkers like sST2, may improve diagnostic precision (44). In addition, recent evidence from Menghoum's study suggests that CA125 represents a promising biomarker of congestion, particularly in HFpEF, further highlighting the need to expand future multi-biomarker strategies (45). Future research should focus on refining biomarker thresholds and establishing their utility in distinguishing HF phenotypes, ultimately enhancing individualized HF management strategies.

There is a deficit in systematic reviews related to the diagnostic role of inflammatory biomarkers in heart failure. A previous meta-analysis that studied CRP, IL-6 and TNF receptor-1 in HFrEF and HFpEF concluded that HFpEF can be differentiated from HFrEF by a higher concentration of IL-6 and lower level of NO (62). However, in our study there is no difference between HFrEF and HFpEF based on inflammatory biomarkers. The results from the meta-analyses of IL-6, hs-CRP, and TNF-alpha levels in heart failure patients compared to healthy controls consistently demonstrate that systemic inflammation is significantly elevated in heart failure, with inflammatory markers showing a strong association with disease severity. However, the high heterogeneity observed in these meta-analyses suggests that further research is needed to clarify the relationship between IL-6, TNF-alpha, and hs-CRP in relation to heart failure subtypes, and to assess whether IL-6, TNF-alpha, and hs-CRP could serve as a reliable biomarkers for disease severity or treatment response.

Studies show that high-sensitivity cardiac troponin and cystatin C are strong predictors of all-cause and cardiovascular mortality (63–65). The importance of multiple biomarker monitoring was described in a recent systematic review, but the inclusion criteria was focused mainly on the acute form of heart failure (66). Rabkin conducted a systematic review with meta-analysis focused on GDF-15, Galectin-3, sST2 and NT-proBNP (67). They came to similar conclusions when compared NT-proBNP levels between HFrEF and HFpEF. However, the meta-analysis of sST2 and GDF-15 yielded slightly differed results. Rabkin reported that sST2 levels were slightly higher in HFrEF, with higher heterogeneity in the studies (*I*^2^ = 55.8%). On the contrary, our meta-analysis found elevated sST2 levels in the HFpEF phenotype, with low heterogeneity (*I*^2^ = 11%). The GDF-15 meta-analysis also differed significantly from Rabkin's observations. Our results demonstrated a statistically significant elevation of GDF-15 in HFrEF, and Rabkin showed no statistical significance, with slightly higher concentration in HFpEF. Further studies are needed to assess sST2 and GDF-15 levels between HF phenotypes, as the small number of studies and limited population sizes may not accurately represent HF phenotypes' nature.

A strength of our study is that our articles include a larger population than previous systematic reviews, making it more statistically reliable. A limitation of our study is that the number of included studies with phenotypically diversified data is not enough to conduct a meta-analysis comparing HF phenotypes for some biomarkers of interest, such as ET-1, Galectin-3, hs-CRP, TNF-alpha, and cystatin C. The varying classifications of heart failure make it challenging to focus only on congestive heart failure cohorts. Some included studies classify patients under general heart failure, resulting in mixed groups that include both congestive and acute heart failure patients.

In conclusion, our comprehensive review reveals the complex role of inflammatory biomarkers (IL-6, TNF-alpha, hs-CRP) in heart failure, demonstrating their variable associations with disease subtypes, comorbidities, and outcomes. These biomarkers show potential as indicators of severity, progression, and treatment response, paving the way for personalized management. Notably, comorbidities significantly influence biomarker concentrations, necessitating a nuanced interpretation, especially when differentiating HFrEF from HFpEF. The lack of HFpEF-specific data highlights an urgent research need. Beyond inflammation, cardiac remodeling and congestion biomarkers such as sST2, Galectin-3, GDF-15, osteopontin, and ET-1 provide valuable prognostic insights, reflecting fibrosis, extracellular matrix degradation, and adverse ventricular remodeling. These markers have demonstrated predictive potential for heart failure progression, hospitalization, and mortality, although their clinical implementation remains limited by variability across studies. Additionally, myocardial injury biomarkers, including hs-TnT, hs-TnI, and Cystatin C, are crucial for assessing myocardial injury and distinguishing between ischemic and non-ischemic heart failure etiologies. Their elevated levels in HFrEF suggests a direct link to cardiomyocyte damage and necrosis, further reinforcing their diagnostic and prognostic relevance. Given the multifaceted pathophysiology of heart failure, a multi-biomarker approach integrating inflammatory biomarkers, biomarkers of cardiac remodeling and congestion, and biomarkers of myocardial injury may enhance diagnostic precision and risk stratification. Future research should focus on refining biomarker panels, establishing optimal cutoff values, and exploring their role in personalized heart failure management. Standardized sampling protocols and longitudinal studies are necessary to validate these findings and optimize clinical application.

## Data Availability

The original contributions presented in the study are included in the article/Supplementary Material, further inquiries can be directed to the corresponding author.

## Supplementary material

Supplementary Figure S1 Risk of bias graph.

Supplementary Figure S2 Risk of bias summary.
