# Supplementary material for: Diagnostic and prognostic value of circulating biomarkers in heart failure
Source: Front Cardiovasc Med. 2025 Sep 24;12:1633164. doi: 10.3389/fcvm.2025.1633164 (PMC12504382; doi:10.3389/fcvm.2025.1633164)
Supplement: Supplementary file 1 [file Datasheet1.pdf]

Supplementary Table 1. Search strategy terms

Search Database	Search terms
Embase	'congestive heart failure'/mj AND ('c reactive protein'/exp OR 'c reactive protein' OR 'c reaction protein' OR 'c-reactive protein' OR 'creactive protein' OR 'crp' OR 'protein, c reactive' OR 'serum c reactive protein' OR 'tumor necrosis factor'/exp OR 'tnf alfa' OR 'tnf alpha' OR 'cachectin' OR 'cachetin' OR 'human recombinant tumour necrosis factor alpha' OR 'mhr 24' OR 'recombinant tumour necrosis factor alpha' OR 'tissue necrosis factor' OR 'tumor necrosis factor' OR 'tumor necrosis factor alfa' OR 'tumor necrosis factor alpha' OR 'tumor necrosis factor-alpha' OR 'tumor necrosis factors' OR 'tumor necrosis serum' OR 'tumour necrosis factor' OR 'tumour necrosis factor alfa' OR 'tumour necrosis factor alpha' OR 'tumour necrosis factor-alpha' OR 'tumour necrosis factors' OR 'tumour necrosis serum' OR 'interleukin 6'/exp OR '26 k protein' OR 'b cell stimulatory factor 2' OR 'b cell stimulating factor 2' OR 'b lymphocyte stimulating factor 2' OR 'beta 2 interferon' OR 'beta2 interferon' OR 'bsf 2' OR 'bsf2' OR 'hepatocyte stimulating factor' OR 'il 6' OR 'interferon beta 2' OR 'interferon beta2' OR 'interleukin 6' OR 'interleukin b' OR 'interleukin hp1' OR 'interleukin-6' OR 'liver cell stimulating factor' OR 'plasmacytoma growth factor' OR 'protein 26k' OR 'interleukin 1beta'/exp OR 'il 1 beta' OR 'il 1beta' OR 'beta interferon inducing 22 k factor' OR 'beta interferon inducing 22k factor' OR 'interferon beta inducing 22k factor' OR 'interleukin 1 beta' OR 'interleukin 1beta' OR 'interleukin-1beta' OR 'interleukin 18' OR 'amino terminal pro brain natriuretic peptide'/exp OR 'galectin 3'/exp OR 'galectin 3' OR 'soluble suppression of tumorigenicity 2'/exp OR 'growth differentiation factor 15'/exp OR 'osteopontin'/exp OR 'spp1 protein' OR 'bone sialoprotein 1' OR 'osteopontin' OR 'protein spp1' OR 'secreted phosphoprotein 1' OR 'myeloperoxidase'/exp OR 'e.c. 1.11.2.2' OR 'myeloperoxidase' OR 'myeloperoxidase a' OR 'peroxidase, myelo' OR 'sestrin'/exp OR 'sesn protein' OR 'sesn proteins' OR 'sestrin' OR 'sestrin family' OR 'sestrin protein' OR 'sestrin proteins' OR 'sestrins' OR 'endothelin 1'/exp OR 'endothelin 1' OR 'endothelin-1' OR 'troponin'/exp OR 'troponin' OR 'troponin complex' OR 'troponin component' OR 'cystatin c'/exp OR 'cystatin 3' OR 'cystatin c' OR 'gamma trace' OR 'neuroendocrine basic polypeptide' OR 'post gamma globulin') AND ('biological marker'/exp OR 'biological marker' OR 'biological markers' OR 'biomarker' OR 'biomarkers' OR 'marker, biological') AND 'human'/de AND 'article'/it
MEDLINE	(congestive heart failure[MeSH Terms]) AND (biomarkers[MeSH Terms])) AND ((c reactive protein[MeSH Terms]) OR (tumor necrosis factor alpha[MeSH Terms]) OR (receptors, tumor necrosis factor[MeSH Terms]) OR (interleukin 6[MeSH Terms]) OR (interleukin 1 beta[MeSH Terms]) OR (interleukin 18[MeSH Terms]) OR (brain natriuretic peptide[MeSH Terms]) OR (galectin 3[MeSH Terms]) OR (sST2[Title]) OR (soluble suppression of tumorogenicity 2[Title]) OR (gdf 15[MeSH Terms]) OR (osteopontin[MeSH Terms]) OR (myeloperoxidase[MeSH Terms]) OR (sestrin[Title]) OR (cystatin c[MeSH Terms]) OR (endothelin 1[MeSH Terms])) AND ((booksdocs[Filter] OR clinicaltrial[Filter] OR randomizedcontrolledtrial[Filter]) AND (humans[Filter]) AND (english[Filter]))
Scopus	AUTHKEY ({chronic heart failure} OR {congestive heart failure} OR {heart failure}) AND AUTHKEY ({biomarker}) AND ({C-reactive protein} OR {tumor necrosis factor-alpha} OR {Interleukin 1} OR {Interleukin 6} OR {Interleukin 18} OR {NTproBNP} OR {Galectin 3} OR {sST2} OR {GDF-15} OR {Osteopontin} OR {Myeloperoxidase} OR {sestrin} OR {Endothelin 1} OR {Troponin}) AND (LIMIT-TO (DOCTYPE , "ar")) AND (LIMIT-TO (LANGUAGE , "English")) AND (LIMIT-TO (EXACTKEYWORD , "Human"))

Supplementary Table 2. Studies assessing inflammatory biomarkers for CHF

Study	Study population	Study design	N, subjects	Subject subgroups (N, Age)	Inflammatory markers	Comorbidities	Conclusion of research
Tromp (2017) (1)	Chronic HF population, Netherlands	Case control study	460	HFrEF (364, 69.6±11.2), HFpEF (96, 74.5±10.0)	TNF-alpha, TNF-alpha-R1a, IL-6, MPO, hs-CRP	Atrial fibrillation (45.4%), diabetes mellitus (29.3%), COPD (28.3%), hypertension (41.5%), anemia (27.8%), myocardial infarction (40.7%)	Levels of hs-CRP were higher in HFpEF compared to HFrEF. There was no significant difference in the concentrations of TNF-alpha, TNF-alpha-R1a, MPO, and hs-CRP.
Boulogne (2017) (2)	Acute and chronic HF population, France	Clinical trial	75	Acute HFrEF (55, 71.0 (63.5–79.0)), Chronic HFrEF (20, 57.0 (48.0–62.5))	TNF-alpha, IL-6, MPO, hs-CRP	Diabetes (25%), COPD (7%), atrial fibrillation (34%)	TNF-alpha, MPO, and hs-CRP levels were significantly higher in acutely decompensated HF compared to chronic HF. IL-6 levels did not show a significant difference between the two cohorts.
Abernethy (2018) (3)	CHF population, USA	Randomized clinical trial	161	Stable HFpEF (83, 72 (65–79)), Acutely decompensated HFpEF (78, 73 (65–79))	TNF-alpha, IL-6, hs-CRP	Atrial fibrillation (60.2%), diabetes mellitus (47.8%), orthopnea (71.1%), hypertension (83.2%)	Compared to stable HFpEF, the acutely decompensated group had higher TNF-alpha, IL-6, and hs-CRP levels.
Almasood (2011) (4)	HF population, Saudi Arabia	Clinical trial	82	HF (82, 49.1±12)	TNF-alpha, IL-6, IL-1 beta	Diabetes (14.6%), hypertension (25.9%)	TNF-alpha and IL-6 were positively correlated with the NYHA class. IL-1 beta concentration was below the detection limit.

Pryds (2019) (5)	Chronic ischemic HF population, Denmark	Controlled clinical trial	42	HF (21, 66.7 ± 9.8), HC (21, 63.1 ± 6.3)	TNF-alpha, IL-6, hs-CRP	Hypertension (52%)	Only NYHA I-II patients with a low comorbidity burden were recruited; therefore, inflammatory biomarkers showed a weak difference between HF and healthy controls. IL-1 beta concentration was below the detection limit and was not reported.
Stanciu (2018) (6)	CHF population, Romania	Clinical trial	62	HFrEF (32, 60±10), HC (30, 57±7)	TNF-alpha, IL-6, IL-1 beta	No comorbidities	CA-125 correlated with coronary sinus IL-1 beta, peripheral venous IL-6, and TNF-alpha levels. IL-1 beta levels were significantly different between the HFrEF and HC cohorts.
Susa (2012) (7)	Chronic systolic HF, Japan	Observational study	186	No cardiac event CHF (123, 57.1±13.6), cardiac event CHF (63, 55.8±17)	TNF-alpha, IL-6, hs-CRP	-	There was no difference in TNF-alpha and IL-6 concentrations between groups with and without cardiac events. However, hs-CRP levels were significantly higher in the event group.
Davarzani (2018) (8)	CHF population, Switzerland	Randomized controlled trial	499	No event CHF during last 19 months (312, 75.1±7.5), One or more events CHF (187, 77.9±7.2)	IL-6, hs-CRP	-	The event CHF cohort had a higher age, NYHA classification, and IL-6 and hs-CRP levels compared to the no-event CHF group.

Everett (2019) (9)	Myocardial infarction population, USA	Randomized controlled trial	10061	HF (385, 66 (59, 73)), HC (9676, 61 (54, 68))	Hs-CRP, IL-6	Diabetes mellitus (40%), Hypertension (80%)	Therapy with an IL-1 beta inhibitor in the myocardial infarction population may reduce HF and HF-related mortality.
Fedacko (2014) (10)	Chronic HF adult population, India	Case control study	366	CHF (116, 51.7±7.5) (HFrEF n=86, HFpEF n=30), HC (250, 52.5±10.5)	TNF-alpha, IL-6	Hypertension	Pro-inflammatory cytokines can be indicators of the causes and severity of CHF. TNF-alpha and IL-6 concentrations are significantly higher in LVEF "<50%" group compared to LVEF ">50%".
Nakamura (2009) (11)	CHF population, Japan	Observational study	118	NYHA I (26, 59.8 ±2.7), NYHA II (26, 59.7±2.4), NYHA III (25, 61.2±2.9), NYHA IV (21, 64.2±2.1), HC (20, 56.8±3.2)	TNF-alpha, hs-CRP	-	TNF-alpha and hs-CRP concentrations increased exponentially with increasing NYHA class.
Niebauer (2005) (12)	HF population, UK	Clinical trial	27	HF (18, 53.6±9.2), HC (9, 51.3±6.9)	TNF-alpha, TNF-SR1, TNF-SR2, IL-6	-	IL-6, TNF-alpha, and TNF-alpha receptor levels are not associated with adverse outcomes.
Richter (2013) (13)	HF population, Austria	Observational study	349	HF survivors (154, 67 (58-77)), HF non-survivors (195, 79 (67-83))	hs-TNF-alpha, MPO	COPD (19.2%), diabetes ,ellitus (40.1%), hypertension (69.1%)	TNF-alpha levels predicted all-cause mortality. Myeloperoxidase levels did not show any significant difference between the two groups and were

							not associated with mortality.
White (2006) (14)	CHF population, Canada	Randomized controlled trial	44	CHF (29, 61.9±2.7), HC (15, 54±3.2)	IL-6, hs-CRP	Hypertension (45%), diabetes (45%)	IL-6 and hs-CRP levels were significantly elevated in the CHF group compared to the healthy control group.
Askevold (2013) (15)	CHF population, Norway	Randomized controlled trial	1455	CHF (1455, 71.8±6.9)	IL-6, sgp130	Hypertension (69%), diabetes mellitus (26%), atrial fibrillation (21.9%), myocardial infarction (63.1%)	sgp130, but not IL-6, was found to be associated with fatal outcomes in ischemic CHF.
Grossman (2001) (16)	CHF population, Brazil	Controlled clinical trial	25	CHF (18, 47±11), HC (7, 38±10)	TNF-alpha, TNF-SR1, TNF-SR2	-	TNF-alpha production predominantly may occur at peripheral site.
Sobieszek (2020) (17)	CHF population, Poland	Clinical trial	66	HF (66, 77±9)	TNF-alpha	Diabetes mellitus (42.4%), Renal failure (37.9%)	There is negative correlation between TNF-alpha against irisin level. TNF-alpha concentration is significantly higher in cathectic CHF group.
Aulin (2022) (18)	Atrial fibrillation with HF, Sweden	Randomized clinical trial	11818	HFrEF (2048, 67.0 (60.0 - 74.0)), HFpEF (2520, 69.0 (61.0 - 74.0)), HC (7250, 70.0 (63.0-76.0))	IL-6	Diabetes mellitus (25%), hypertension (86.9%)	Inflammatory biomarkers improve the identification of the risk of developing or worsening HF.
Kaye (2022) (19)	HF population, Australia	Observational study	34	HFpEF (20, 70±2), HC (14, 53±2)	IL-6	Hypertension (65%), atrial fibrillation (55%), diabetes (25%), coronary disease (20%)	IL-6 levels correlated with BMI and age and were significantly higher in the HFpEF group compared to the HC group.

Pandhi (2022) (20)	Chronic HF population, Europe (Netherlands)	Observational study	550	No congestion CHF (408, 66.6 ± 11.7), Severe congestion CHF (142, 69.6 ± 12.8)	IL-6	Diabetes (30%), hypertension (64%), atrial fibrillation (42%), COPD (14.5), renal disease (30%)	IL-6 is significantly elevated in patients with severe congestive CHF.
Wang (2014) (21)	HF population, China	Clinical trial	1812	HF (1364, 57.9±14.7), HC (448, 57.1±18.3)	IL-6	Hypertension (30.8%)	IL-6 independently was weak predictor of mortality.
Cakmak (2015) (22)	HF population, Turkey	Case control study	57	HFrEF (42, 56.57 ± 10.35), HC (15, 51.78 ± 3.9)	hs-CRP	Diabetes mellitus (40.4%), chronic renal disease (14.3%), hypertension (73.8%), hyperlipidemia (69%), atrial fibrillation (14.3%), COPD (14,3%), coronary artery disease (35.7%)	Some miRNA has positive correlation with hs-CRP cytokine levels.
De Marco (2021) (23)	HF population, USA/Canada /Russia	Randomized clinical trial	248	HFpEF with diabetes mellitus (116, 69±9.3), HFpEF without diabetes mellitus (132, 74.3±9.7)	hs-CRP	Cardiovascular disease (55.24%), myocardial infarction (21.37%), hypertension (93.55%), stroke (7.66%), COPD (11.3%), asthma (12.5%), dyslipidemia (78.22%), atrial fibrillation (49.6%)	The inflammatory biomarker high-sensitivity C-reactive protein (hs-CRP) was higher in patients with diabetes.
Djoussé (2014) (24)	HF population, USA	Randomized clinical trial	924	HF (462, 58.2±8.1), HC (462, 58.2±8.1)	hs-CRP	Atrial fibrillation (5.4%), diabetes (6.9%), hypertension (37.5%)	There is no significant difference in hs-CRP concentration between the HF and HC groups.
Do e (2013) (25)	Chronic HF population, Japan	Clinical trial	160	CHF (130, 58±1), HC (30, 54±2)	hs-CRP	-	hs-CRP concentration was significantly higher in CHF compared to HC. Rho-kinase activity was not

							associated with hs-CRP levels.
Dudek (2023) (26)	Chronic HF population, Poland	Observational study	110	CHF sST2<45.8 (74, 53±11.7), CHF sST2>45.8 (36, 53.4±11)	hs-CRP	Atrial fibrillation (16.4%), diabetes mellitus type II (25.5%), arterial hypertension (44.6%), thyroid disease (19.1%), COPD (12.7%)	hs-CRP levels increase with rising sST2 levels. The study showed that the combination of high hs-CRP (>6.4 mg/L) and elevated sST2 (>47.6 ng/mL) significantly increased the risk of mortality.
Dubrock (2018) (27)	HFpEF population, USA	Clinical trial	214	Normal hs-CRP HFpEF (93, 70 (65-79)), high hs-CRP HFpEF (121, 67 (61-76))	hs-CRP	Diabetes mellitus (43%), hypertension (85%), ischemic heart disease (38%), Atrial fibrillation (51%), COPD (20%), anemia (40%).	Correlation with hs-CRP level and NYHA functional classification wasn't observed. High level of hs-CRP was associated with comorbidity burden, but 40% of HFpEF patients had hs-CRP at a normal range. There were no significant associations between CRP and NT-proBNP.
Florea (2016) (28)	HF population, USA	Randomized controlled trial	3519	HFrEF (3198, 62±11), HF with improved EF (321, 61±11)	hs-CRP	Diabetes mellitus (25%), hypertension (6.5%), atrial fibrillation (11%), COPD (11.8%)	There was no difference in high-sensitivity C-reactive protein levels between the HFrEF and HFiEF groups.
Ge (2023) (29)	HF population, China	Observational study	276	HF (128, 61 (52-68)), HC (148, 61 (50-66))	hs-CRP	Hypertension (50%), diabetes mellitus (35.9%), hypercholesterolemia (10.9%), atrial fibrillation (25.8%), COPD (2.3%),	hs-CRP and NT-proBNP concentrations were significantly higher in HF compared to HC.

						myocardial infarction (48.4%), anemia (3.9%)	
Gravning (2014) (30)	Chronic HF population, AstraZeneca	Randomized controlled trial	2490	HFrEF (2490, 71.8)	hs-CRP	Hypertension (70%), diabetes mellitus (26%), atrial fibrillation (21%)	Elevated hs-CRP levels are associated with hs-cTnT levels.
Loncar (2010) (31)	Chronic HF population, Serbia	Observational study	95	CHF (75, 68±7), HC (20, 67±7)	hs-CRP	Myocardial infarction (65%), atrial fibrillation (27%), hypertension (63%)	hs-CRP protein levels showed no significant difference between the healthy control and CHF cohorts.
Loncar (2012) (32)	Chronic HF population, Serbia	Randomized controlled trial	398	HFrEF (325, 72±5), HFpEF (73, 72±5)	hs-CRP	Diabetes (27%), hypertension (80.6%), coronary artery disease (63.6%)	hs-CRP protein levels showed no significant difference between the HFrEF and HFpEF groups.
Mohebi (2022a) (33)	HF population, USA	Observational study	1235	Stage A HF (77, 57.3±8.9), stage B HF (733, 66±11.3), stage C/D HF (425, 68.8±11.5)	hs-CRP	Type 2 diabetes (25.58%), hypertension (75.46%), hyperlipidemia (66.8%), CKD (13%), COPD (17.73%), myocardial infarction (23.24%), atrial fibrillation (18.62%)	In HF stage C/D, hs-CRP was associated with CV death and HF hospitalization.
Rathcke (2010) (34)	CHF population, Denmark	Observational study	311	CHF (194, 71±7.9), HC (117, 69.3±10.3)	hs-CRP	Diabetes mellitus (25.3%)	hs-CRP level is significantly higher in the CHF group compared to the healthy control group.
Ren (2023) (35)	HF population, China	Observational study	964	Event HF (232, 68±14), no-event HF (732, 62±14.4)	hs-CRP	Hypertension (57.8%), diabetes mellitus (35%), hyperlipidemia (32.4%), chronic kidney disease (18.2%), myocardial infarction (16.2%),	hs-CRP level is significantly higher in event HF group compared to no-event HF.

						stroke (13.7%), atrial fibrillation (28.8%)	
Teramoto (2024) (36)	Chronic HF, Japan	Randomized controlled trial	942	CHF under 50 years (73, 44 (39-47)), CHF 50-59 years (158, 56 (54-58)), CHF 60-69 years (296, 65.5 (63-80)), CHF 70-79 years (415, 75 (72-77))	hs-CRP	Diabetes (47.7%), dyslipidaemia (52%), ischaemic heart disease (49.3%), cardiomyopathy (25.5%), atrial fibrillation (41.5%)	hs-CRP was not correlated with increasing age. Adding GDF-15 to the fully adjusted model, which included demographics and other biomarkers (NT-proBNP, troponin, and CRP), showed a marginal improvement in model performance for HF hospitalization or all-cause death but not for major adverse cardiac events (MACE).
Tymińska (2019) (37)	Population without HF, Poland	Observational study	104	Without HF at 1 year (54, 58(43-67.3)), With HF at 1 year (50, 64(57-70.8))	hs-CRP	Hypertension (64%), atrial fibrillation (10%), diabetes (30%), chronic kidney disease (22%), COPD (6%)	Baseline hs-CRP levels are higher in the LVEF <50% group compared to the LVEF >50% group. The average hs-CRP level is higher in the HF group compared to those without HF during the one-year follow-up.
Wołowiec (2023) (38)	HF population, Poland	Observational study	120	Death HFrEF (44, 69.91±13.74), survived HFrEF (76, 55.53±12.8)	hs-CRP	-	hs-CRP levels showed no difference between the deceased HFrEF group and the surviving HFrEF group.
Wołowiec (2024) (39)	HF population, Poland	Observational study	122	Stable HFrEF (52, 51.58±9.16), HFrEF with	hs-CRP	Diabetes mellitus (40.9%), hypertension (53.3%), atrial fibrillation (49.1%)	Baseline hs-CRP concentration was higher in the exacerbation HFrEF

				exacerbation (70, 67.93±14.38)			group compared to the stable HFrEF group.
Yu (2024) (40)	HF population, China	Observational study	116	HF (80, 60.9±9.62), HC (36, 54.8±8.88)	hs-CRP	Diabetes mellitus (28.75%), hypertension (72.5%), atrial fibrillation (30%), chronic renal failure (13.75%), myocardial infarction (17.5%), cerebral stroke (21.25%)	hs-CRP level is significantly higher in the HF group compared to healthy controls.

Supplementary Table 3. Studies assessing cardiac remodeling and congestion biomarkers for CHF

Study	Study population	Study design	N, subjects	Subject subgroups (N, Age)	Cardiac remodeling/congestion markers	Comorbidities	Conclusion of research
Tromp (2017) (1)	Chronic HF population, Netherlands	Case control study	460	HFrEF (364, 69.6±11.2), HFpEF (96, 74.5±10.0)	ST-2, GDF-15, Gal-3, ET-1, NT-proBNP, osteopontin	Atrial fibrillation (45.4%), diabetes mellitus (29.3%), COPD (28.3%), hypertension (41.5%), anemia (27.8%), myocardial infarction (40.7%)	NT-proBNP concentration is significantly lower in patients with HFpEF compared to those with HFrEF. There wasn't a significant difference in Galectin-3, ST2, osteopontin, GDF-15, or Endothelin-1 concentrations between phenotypes.
Florea (2016) (28)	HF population, USA	Randomized controlled trial	3519	HFrEF (3198, 62±11), HF with improved EF (321, 61±11)	sST2, GDF-15, Gal-3, ET-1, NT-proBNP	Diabetes mellitus (25%), hypertension (6.5%), atrial fibrillation (11%), COPD (11.8%)	HFiEF group has lower NT-proBNP, sST2, Galectin-3, Endothelin-1 and GDF-15 concentrations compared to HFrEF group.
Mohebi (2022a) (33)	HF population, USA	Observational study	1235	Stage A HF (77, 57.3±8.9), stage B HF (733, 66±11.3), stage C/D HF (425, 68.8±11.5)	sST2, Gal-3, ET-1, NT-proBNP	Type 2 diabetes (25.58%), hypertension (75.46%), hyperlipidemia (66.8%), CKD (13%), COPD (17.73%), myocardial infarction (23.24%), atrial fibrillation (18.62%)	In the earlier stages (Stage A/B) of HF, NT-proBNP**,** Endothelin-1**, and** Galectin-3 are significant predictors of HF hospitalization and cardiovascular (CV) death. sST2 is a reliable biomarker for predicting adverse outcomes in patients

							with advanced heart failure (Stage C/D).
Davarzani (2018) (8)	CHF population, Switzerland	Randomized controlled trial	499	No event CHF during last 19 months (312, 75.1±7.5), One or more events CHF (187, 77.9±7.2)	sST2, GDF-15, NT-proBNP, osteopontin	-	The event CHF cohort has a higher age, NYHA class, and GDF-15, sST2, and NT-proBNP levels compared to the no-event CHF group.
Ehteshami-Afshar (2021) (41)	Chronic HF population, Novartis Pharmaceuticals	Randomized controlled trial	8399	HFrEF without COPD (7319, 63.3±11.6), HFrEF with COPD (1080, 67.4±9.5)	ST2, GDF-15, Gal-3, NT-proBNP	Hypertension (70.72%), atrial fibrillation (36.8%), myocardial infarction (43.26%), diabetes mellitus (34.48%), stroke (8.6%)	GDF-15 and NT-proBNP concentrations were significantly higher in HFrEF with COPD group. COPD was not associated with ST2 and Galectin-3.
Boulogne (2017) (2)	Acute and chronic HF population, France	Clinical trial	75	Acute HFrEF (55, 71.0 (63.5–79.0)), Chronic HFrEF (20, 57.0 (48.0–62.5))	sST2, GDF-15, Gal-3	Diabetes (25%), COPD (7%), atrial fibrillation (34%)	GDF-15 levels moderately decreased in the CHF cohort compared to AHF. sST2 and Galectin-3 levels did not differ significantly between the two cohorts and were not associated with adverse outcomes.
De Marco (2021) (23)	HF population, USA/Canada /Russia	Randomized clinical trial	248	HFpEF with diabetes mellitus (116, 69±9.3), HFpEF without diabetes mellitus (132, 74.3±9.7)	sST2, Gal-3, NT-proBNP	Cardiovascular disease (55.24%), myocardial infarction (21.37%), hypertension (93.55%), stroke (7.66%), COPD (11.3%), asthma (12.5%), dyslipidemia (78.22%), atrial fibrillation (49.6%)	Galectin-3 levels were significantly higher in the HFpEF with diabetes group. sST2 and NT-proBNP concentrations showed no difference between groups.

Gaggin (2016) (42)	Chronic HF population, USA	Randomized controlled trial	108	HFrEF with cachexia (20, -), HFrEF without cachexia (88, -)	sST2, ET-1, NT-proBNP	-	Patients who developed cachexia during follow-up had higher concentrations of sST2, ET-1, and NT-proBNP compared to those with HF without cachexia.
Jungbauer (2014) (43)	Chronic HF population, Germany	Clinical trial	233	CHF (149, 61.8±11.6), HC (84, 42.9±8.3)	GDF-15, Gal-3, NT-proBNP	-	The multimarker panel consisting of NT-proBNP, hs-TnT, TIMP-1, GDF-15, and IBP-4 showed improved prognostic information compared to the combination of NT-proBNP and hs-TnT alone.
Tymińska (2019) (37)	Population without HF, Poland	Observational study	104	Without HF at 1 year (54, 58(43-67.3)), With HF at 1 year (50, 64(57-70.8))	sST2, Gal-3, NT-proBNP	Hypertension (64%), atrial fibrillation (10%), diabetes (30%), chronic kidney disease (22%), COPD (6%)	Galectin-3 negatively correlates with LV end-diastolic volume and was the primary predictor of the endpoint. Galectin-3 and sST2 were not associated with diastolic dysfunction parameters; therefore, they cannot be used for the diagnosis of HFpEF. NT-proBNP was higher in the LVEF <50% group compared to the LVEF >50% group.
Ahmad (2023) (44)	Chronic HF population, Pakistan	Observational study	139	Ischemic chronic HF (56, 66.8±4.2), Non-ischemic chronic	sST2, Gal-3	-	Galectin-3 levels are higher in the ischemic CHF group, but there is no association

				HF (24, 66.8±3.9), HC (59, 63.5±3.6)			between Galectin-3 and frailty in CHF. sST2 levels were observed to be high in both ischemic and non-ischemic cohorts and correlated with cardiac contractile dysfunction, LV remodeling, and hypertrophy.
Lupón (2013) (45)	Chronic HF population, Spain	Clinical trial	876	Alive HF (565, 66.1 (56.5-74.3)), Deceased HF (311, 75.6 (69.9-81.0))	hs-ST2, NT-proBNP	Hypertension (61.2%), diabetes mellitus (35.8%), chronic obstructive lung disease (16.9%), sleep apnoea (4.5%), atrial fibrillation (16.7%)	NT-proBNP added to multimarker analysis with hs-cTnT and hs-ST2 does not improve prognostic accuracy. The combination of hs-cTnT and hs-ST2 identified more decedents during follow-up compared to the combination of NT-proBNP, hs-cTnT, and hs-ST2.
Menghoum (2024) (46)	HF population, Belgium	Observational study	164	HFpEF (139, 78±8), HC (25, 77±5)	sST2, NT-proBNP	Atrial fibrillation (60%), coronary artery disease (33%), myocardial infarction (12%), COPD (9%), sleep apnoea (13%)	NT-proBNP and sST2 levels were significantly higher in HFpEF compared to HC. CA 125 and NT-proBNP levels were independently associated with long-term all-cause mortality.
Roy (2020) (47)	HF population, Belgium	Clinical trial	174	HFpEF (143, 78±8), HC (31, 75±6)	sST2, NT-proBNP	Atrial fibrillation (62%), chronic obstructive pulmonary disease (10%), sleep apnoea (13%)	With higher fibroblast growth factor 23 (FGF-23) concentrations, levels of sST2 and NT-

							proBNP increase. sST2 and NT-proBNP levels are significantly higher in HFpEF compared to HC.
Mendez-Fernandez (2020) (48)	HF population, Spain	Observational study	311	HFmrEF (90, 67±14), HFpEF (221, 73±12)	GDF-15, NT-proBNP	Hypertension (77%), diabetes mellitus (37%), dyslipidemia (48%), atrial fibrillation (49%)	GDF-15 emerged as an independent predictor of all-cause mortality in patients with LVEF >40%. It was superior in prognostic assessments of the HFpEF and HFmrEF cohorts.
Teramoto (2024) (36)	Chronic HF, Japan	Randomized controlled trial	942	CHF under 50 years (73, 44 (39-47)), CHF 50-59 years (158, 56 (54-58)), CHF 60-69 years (296, 65.5 (63-80)), CHF 70-79 years (415, 75 (72-77))	GDF-15, NT-proBNP	Diabetes (47.7%), dyslipidaemia (52%), ischaemic heart disease (49.3%), cardiomyopathy (25.5%), atrial fibrillation (41.5%)	GDF-15 is a powerful determinant of cardiovascular endpoints, in addition to other established risk factors, particularly in patients around 70 years old. In patients aged 60–70, NT-proBNP showed a strong association with diabetes as a comorbid condition.
Claus (2020) (49)	CHF population, France/Germany	Observational study	153	CHF (73, 64.3±16.2), HC (80, 56.6±18.2)	GDF-15, NT-proBNP	-	A multi-marker model combining clinical parameters, NT-proBNP, GDF-15, and cNEP activity provided the best discrimination between CHF and healthy control cohorts.

Aulin (2022) (18)	Atrial fibrillation with HF, Sweden	Randomized clinical trial	11818	HFrEF (2048, 67.0 (60.0 - 74.0)), HFpEF (2520, 69.0 (61.0 - 74.0)), HC (7250, 70.0 (63.0-76.0))	GDF-15, NT-proBNP	Diabetes mellitus (25%), hypertension (86.9%)	GDF-15 improves the identification of the risk of developing or worsening HF. NT-proBNP was more strongly associated with adverse events in the HFrEF group compared to HFpEF.
Richter (2013) (13)	HF population, Austria	Observational study	349	HF survivors (154, 67 (58-77)), HF non-survivors (195, 79 (67-83))	GDF-15, NT-proBNP	COPD (19.2%), diabetes mellitus (40.1%), hypertension (69.1%)	A multi-biomarker score consisting of NT-proBNP, fractalkine, hepatocyte growth factor (HGF), GDF-15, soluble apoptosis-stimulating fragments (sFAS), and soluble tumor necrosis factor-related apoptosis-inducing ligand (sTRAIL) demonstrated that a multi-biomarker approach reflecting the multisystemic character of HF is superior to a comprehensive conventional prediction algorithm that includes clinical variables and NT-proBNP.
Poglajen (2019) (50)	Chronic HF population (Slovenia)	Clinical trial	61	Ischemic cardiomyopathy CHF (19, 55±8), nonischemic dilated	Gal-3, NT-proBNP	-	Galectin-3 and NT-proBNP levels did not reveal a difference between ischemic and non-ischemic dilated

				cardiomyopathy (42, 51±10)			cardiomyopathy cohorts.
Dubrock (2018) (27)	HF population, USA	Clinical trial	214	Normal hs-CRP HFpEF (93, 70 (65-79)), high hs-CRP HFpEF (121, 67 (61-76))	ET-1, NT-proBNP	Diabetes mellitus (43%), hypertension (85%), ischemic heart disease (38%), Atrial fibrillation (51%), COPD (20%), anemia (40%).	No correlation was observed between hs-CRP levels and NYHA functional classification. A high level of hs-CRP was associated with comorbidity burden, but 40% of HFpEF patients had hs-CRP within the normal range. There were no significant associations between CRP and NT-proBNP. In both bivariate and multivariate analyses, CRP was associated with higher levels of endothelin-1.
Pandhi (2022) (20)	Chronic HF population, Europe (Netherlands)	Observational study	550	No congestion CHF (408, 66.6 ± 11.7), Severe congestion CHF (142, 69.6 ± 12.8)	ET-1, NT-proBNP	Diabetes (30%), hypertension (64%), atrial fibrillation (42%), COPD (14.5), renal disease (30%)	Endothelin-1 and NT-proBNP are significantly elevated in patients with severe congestive CHF.
Wright (2009) (51)	HF population, New Zealand	Randomized controlled trial	299	HF (74, 74±11), HC (225, 72±11)	ET-1, NT-proBNP	-	Endothelin-1 increases with urocortin levels. When NT-proBNP results were considered alone, plasma levels in the upper tertile carried a 58% chance of a final diagnosis of heart failure. When both NT-proBNP and

							urocortin levels were in the upper tertile, the likelihood rose to 75%, and half of all patients with heart failure exhibited top-tertile levels for both peptides.
Akiyama (2020) (52)	HF population, France	Observational study	59	HFpEF (34, 83 (76–89)), HFrEF (25, 63 (57–76))	sST2	-	No difference in sST2 levels was observed between HFrEF and HFpEF groups.
Bahuleyan (2018) (53)	HF population, India	Observational study	141	HF without adverse outcome (84, 59.5±10.3), HF with adverse outcome (57, 61.6±10.7)	sST2	Diabetes mellitus (72.3%), hypertension (59.6%), dyslipidemia (51.1%), coronary artery disease (55.3%)	The baseline concentration of sST2 was significantly higher among patients with adverse outcomes compared to those without adverse outcomes.
Crnko (2020) (54)	Chronic HF population, Netherlands	Observational study	32	CHF (16, 59±13), HC (16, 54±16)	sST2	Diabetes mellitus (31.3%), myocardial infarction (25%), atrial fibrillation (37.5%)	In the vast majority of subjects, sST2 concentration peaked in the afternoon, with the lowest levels occurring at night. Understanding this diurnal variation will improve the use of sST2 as a prognostic biomarker.
Firouzabadi (2020) (55)	HF population, Iran	Observational study	66	HF (44, 66±11), HC (22, 65.2±10.2)	sST2	Diabetes mellitus (40.9%), atrial fibrillation (11.4%), hyperlipidemia (54.5%), stroke (4.5%)	There was no association between HF and HC cohorts based on sST2 concentration.
Gruson (2014) (56)	HF population, Belgium	Observational study (Letter to editor)	137	HFrEF NYHA II-IV (137, 67±14)	sST2	-	In a backward stepwise multivariate COX analysis

							including age, EF, eGFR, sST2, BNP, NT-proBNP, and proBNP levels, only sST2 remained significantly associated with CV death. sST2 was the strongest predictor of long-term CV death in systolic HF.
Andersson (2005) (57)	CHF population, Sweden	Clinical trial	30	CHF (15, 75 (65-81), HC (15, 75)	ET-1	Atrial fibrillation (33.3%), other comorbidities excluded	Heart failure patients exhibit reduced vasodilation in response to endothelin-1, primarily due to a general decline in vascular reactivity and increased vascular stiffness. Despite elevated endothelin-1 levels, endothelin A-mediated vasodilation is significantly diminished, while endothelin B receptor responses remain unchanged.
Galindo-Fraga (2003) (58)	HF population, Mexico	Observational study	43	NYHA I (24, 57±16), NYHA II (15, 65±16), NYHA III (4, 48±22)	ET	Diabetes mellitus (50%), chronic renal failure (14%)	There was a significant difference in endothelin levels between NYHA I and II, and between NYHA I and III, but no difference between NYHA II and III.
Behnes (2013) (59)	CHF population, Germany	Clinical trial	401	Acute CHF (122, 73), no acute CHF (279, 65)	osteopontin	Atrial fibrillation (22%), coronary artery disease (32%),	Osteopontin independently predicted all-cause

						myocardial infarction (22%), valvular heart disease (29%), COPD (8%), chronic kidney disease (17%)	mortality and aCHF-related rehospitalization at 1 and 5 years. Compared to NT-proBNP, osteopontin had superior prognostic value, particularly in aCHF patients and for predicting aCHF-related rehospitalization.
Coculescu (2019) (60)	Chronic HF population, Romania	Observational study	91	CHF (91, 54.55) subgrouped by NYHA classification	osteopontin	-	Higher osteopontin serum levels in men suggest more frequent and intense myocardial remodeling in DHF. Since osteopontin levels correlate with symptom severity, they should be used not only as a biomarker for heart failure but also for prognostic assessment.
Chiu (2024) (61)	HF population, USA	Randomized controlled trial	212	NT-proBNP/cGMP Q1 (53, 62 (55-67)), Q2 (53, 68 (63-76)), Q3 (53, 73 (67-80)), Q4 (53, 73 (65-79))	Gal-3	Hypertension (84.9%), diabetes (42.9%), atrial fibrillation (51.4%), myocardial infarction (28.3%), COPD (28.3%)	LVEF was not associated with the NT-proBNP/cGMP ratio. Patients with a higher NT-proBNP/cGMP ratio had higher Galectin-3 levels. hs-CRP was associated with NT-proBNP levels alone but not with the NT-proBNP/cGMP ratio.
Gocer (2019) (62)	HF population, Turkey/Kyrgyzstan	Observational study	100	NYHA I-IV (100, 41.1)	Gal-3	-	Plasma Galectin-3 concentrations for

							different NYHA classes—mild, moderate, and severe HF—were proposed to be 100–460, 460–1170, and >1170 pg/mL, respectively.
Pryds (2019) (5)	Chronic ischemic HF population, Denmark	Controlled clinical trial	42	HF (21, 66.7 ± 9.8), HC (21, 63.1 ± 6.3)	Gal-3	Hypertension (52%)	Galectin-3 level higher in HF population compared to healthy control.
AbouEzzeddine (2017) (63)	HF population, USA	Clinical trial	174	Low ST2 HF (58, 67 (61-74)), Mid ST2 HF (58, 71 (64-79)), High ST2 HF (58, 69 (63-77))	NT-proBNP	Hypertension (83.9%), atrial fibrillation (48.85%), COPD (18.4%), diabetes mellitus (40.8%)	According to the analysis, patients with higher ST2 levels were more likely to have diabetes mellitus, hypertension, or atrial fibrillation/flutter. Patients with higher ST2 levels also had more congestion and higher NT-proBNP levels.
Mohebi (2022b) (64)	HF population, USA	Observational study	794	HF duration <12 months (178, 61.8±13.1), HF 12-24 months (80, 65.2±14.6), HF 24-60 months (185, 64.3±12.7), HF >60 months (351, 67.3±10.7)	NT-proBNP	Hypertension (88%), stroke (10.8%), myocardial infarction (41.4%), diabetes mellitus (45.5%), atrial fibrillation (33.75%)	NT-proBNP levels have not shown a significant difference based on HF duration.
Abernethy (2018) (3)	CHF population, USA	Randomized clinical trial	161	Stable HFpEF (83, 72 (65–79)), Acutely decompensated HFpEF (78, 73 (65–79))	NT-proBNP	Atrial fibrillation (60.2%), diabetes mellitus (47.8%), orthopnea (71.1%), hypertension (83.2%)	NT-proBNP levels are significantly higher in acutely decompensated HFpEF compared to stable HFpEF.

Askevold (2013) (15)	CHF population, Norway	Randomized controlled trial	1455	CHF (1455, 71.8±6.9)	NT-proBNP	Hypertension (69%), diabetes mellitus (26%), atrial fibrillation (21.9%), myocardial infarction (63.1%)	NT-proBNP levels are higher in the highest quartile of sgp130. Area under the curve analyses favored NT-proBNP as the best single marker for all endpoints and also demonstrated the limited isolated discriminatory power of sgp130.
Bai (2012) (65)	HF population, China	Observational study	220	HFrEF (67, 51±12.6), HFmrEF & HFpEF (66, 56.3±11.5), HC (87, -)	NT-proBNP	Hypertension (37%), diabetes mellitus (21.7%), myocardial infarction (53%), coronary arterial disease (55%)	NT-proBNP was significantly higher in the HFrEF group compared to the HFpEF group.
Brankovic (2019) (66)	Chronic HF population, Netherlands	Clinical trial	263	Composite endpoint CHF (70, 69±13), no composite endpoint CHF (193, 66±12)	NT-proBNP	Atrial fibrillation (40%), myocardial infarction (36%), diabetes (31%), hypercholesterolemia (36%), hypertension (46%), COPD (12%)	Hospitalization for acute or worsened HF was defined as admission due to an exacerbation of HF symptoms, in combination with two or more of the following: BNP or NT-proBNP >3× the upper limit of normal, or signs of worsening HF.
Chenevier-Gobeaux (2008) (67)	CHF population, France	Observational study	253	65-85 years old CHF (144, 75±6), >85 85 years old CHF (109, 90±4)	NT-proBNP	Hypertension (51.8%), coronary artery disease (40%), COPD (23.3%)	In patients older than 85 years, NT-proBNP levels are naturally higher, and the "grey zone" (uncertain diagnosis range) is wider (1,750–6,000 pg/mL) compared to

							650–3,000 pg/mL in CHF patients aged 65–85 years.
Cristóbal (2023) (68)	HF population, Spain	Observational study	168	HFrEF (134, 71 (61-78)), HFpEF (134, 78 (71-82))	NT-proBNP	-	NT-proBNP is a strong prognostic biomarker for survival in both HFrEF and HFpEF groups.
Drum (2017) (69)	HF population, Singapore	Observational study	657	HFrEF (219, 64.7±12), HFpEF (219, 68.2±11.2), HC (219, 65±9)	NT-proBNP	Hypertension (75.8%), diabetes mellitus (57.5%), atrial fibrillation (26%)	TB4 predicts mortality independently of clinical risk factors and NT-proBNP.
Dudek (2023) (26)	Chronic HF population, Poland	Observational study	110	CHF sST2<45.8 (74, 53±11.7), CHF sST2>45.8 (36, 53.4±11)	NT-proBNP	Atrial fibrillation (16.4%), diabetes mellitus type II (25.5%), arterial hypertension (44.6%), thyroid disease (19.1%), COPD (12.7%)	Higher sST2 concentrations are associated with higher NT-proBNP levels. sST2 protein concentration is an independent risk factor for all-cause mortality in patients with stable HF with reduced left ventricular ejection fraction.
Ekure (2011) (70)	CHF population, Nigeria	Observational study	56	CHF (28, 48.5±62.24 months), HC (28, 48.72± 62.15 months)	NT-proBNP	-	Plasma NT-proBNP levels are significantly higher in children with congestive heart failure compared to healthy controls. A high NT-proBNP threshold was identified at 951 pg/mL.
Fröhling (2024) (71)	Chronic HF population, Germany	Observational study	146	CHF (105, 64±17), acutely decompensated HF (41, 69±23.5)	NT-proBNP	Arterial hypertension (59%), dyslipedimia (42%), diabetes mellitus (25.7%)	NT-proBNP levels were significantly higher in the acute decompensated HF

							group compared to the CHF group.
Gao (2019) (72)	Chronic HF population, China	Observational study	344	HFrEF (168, 57.25±14.92), HC (176, 55.11±13.44)	NT-proBNP	Myocardial infarction (31%), diabetes mellitus (23.8%), hypertension (39.3%)	CTRP3 and CTRP9 levels were negatively correlated with NT-proBNP. After a 36-month follow-up, and after adjusting for age, LVEF, and NT-proBNP, we observed that CTRP3 or CTRP9 levels below the 25th percentile were predictors of total mortality and hospitalization.
Gohar (2017) (73)	HF population, Singapore	Observational study	1096	HFrEF (853, 60.2±11.8), HFpEF (243, 68.3±11.4)	NT-proBNP	Coronary artery disease (52.5%), atrial fibrillation (22.5%), hypertension (71.9%), diabetes mellitus (57%)	NT-proBNP does not provide additional prognostic value to the clinical model in HFpEF, as hsTnI and hsTnT do.
Gravning (2014) (30)	Chronic HF population, AstraZeneca	Randomized controlled trial	1245	HFrEF hs-cTnT<14 (629, 70.3±6.5), HFrEF hs-cTnT>14 (616, 73.3±6.9)	NT-proBNP	Hypertension (70%), diabetes mellitus (26%), atrial fibrillation (21%)	The introduction of hs-cTnT levels into the multivariable model (including hs-CRP and NT-proBNP) significantly improved prognostic discrimination for adverse events in HF.
Loncar (2010) (31)	Chronic HF population, Serbia	Observational study	95	CHF (75, 68±7), HC (20, 67±7)	NT-proBNP	Myocardial infarction (65%), atrial fibrillation (27%), hypertension (63%)	Receptor activator of nuclear factor-κB ligand (sRANKL) is significantly associated with serum NT-proBNP and adiponectin, regardless

							of BMI and renal function.
Loncar (2012) (32)	Chronic HF population, Serbia	Randomized controlled trial	398	HFrEF (325, 72±5), HFpEF (73, 72±5)	NT-proBNP	Diabetes (27%), hypertension (80.6%), coronary artery disease (63.6%)	NT-proBNP concentration was significantly higher in the HFrEF group compared to the HFpEF group.
Maeder (2013) (74)	Chronic HF population, Switzerland	Randomized controlled trial	622	HFrEF (499, 76.1±7.5), HFpEF (123, 80.1±7)	NT-proBNP	Hypertension (74.3%), diabetes (35.7%), stroke (15.8%), COPD (20%), peripheral arterial occlusive disease (20%)	NT-proBNP concentration was significantly higher in the HFrEF group compared to the HFpEF group.
Obokata (2019) (75)	HF population, USA	Observational study	58	HFpEF (38, 69±11), HC (20, 62±11)	NT-proBNP	Hypertension (92%), coronary disease (39%), diabetes (39%), atrial fibrillation (18%)	NT-proBNP concentration was significantly higher in the HFpEF group compared to the healthy control group.
Rathcke (2010) (34)	CHF population, Denmark	Observational study	311	CHF (194, 71±7.9), HC (117, 69.3±10.3)	NT-proBNP	Diabetes mellitus (25.3%)	NT-proBNP concentration was significantly higher in the CHF group compared to the healthy control group. A weak correlation was found between YKL-40 and NT-proBNP concentration.
Sobieszek (2020) (17)	CHF population, Poland	Clinical trial	66	HF (66, 77±9)	NT-proBNP	Diabetes mellitus (42.4%), Renal failure (37.9%)	NT-proBNP concentration is significantly higher in cathectic CHF group.
Stanciu (2018) (6)	Chronic HF population, Romania	Clinical trial	62	HFrEF (32, 60±10), HC (30, 57±7)	NT-proBNP	No comorbidities	A direct comparison with NT-proBNP revealed that CA-125 is at least as reliable as

							NT-proBNP in the diagnosis of CHF.
Thibodeau (2022) (76)	HF population, USA	Observational study	153	No congestion HF (65, 61 (50-69)), Mild congestion (35, 59 (50-65)), moderate-severe congestion HF (53, 65 (49-69))	NT-proBNP	Diabetes (40.5%), hypertension (60.8%)	Clinical congestion was associated with a higher frequency of the specific combination of elevated NT-proBNP and hs-troponin T levels.
van Wezenbeek (2018) (77)	HF population, USA	Observational study	166	HFrEF (109, 57±10), HFpEF (57, 53±9)	NT-proBNP	-	NT-proBNP and hs-CRP independently predict peak VO_2 in HF.
Wang (2014) (21)	HF population, China	Clinical trial	1812	HF (1364, 57.9±14.7), HC (448, 57.1±18.3)	NT-proBNP	Hypertension (30.8%)	NT-proBNP concentration was significantly higher in the HFpEF group compared to the healthy control group.
Wołowiec (2023) (38)	HF population, Poland	Observational study	120	Death HFrEF (44, 69.91±13.74), survived HFrEF (76, 55.53±12.8)	NT-proBNP	-	Plasma catestatin (CST) correlated with NT-proBNP levels. NT-proBNP predicted all-cause death in the HF population.
Wołowiec (2024) (39)	HF population, Poland	Observational study	122	Stable HFrEF (52, 51.58±9.16), HFrEF with exacerbation (70, 67.93±14.38)	NT-proBNP	Diabetes mellitus (40.9%), hypertension (53.3%), atrial fibrillation (49.1%)	Baseline NT-proBNP concentration was higher in the exacerbation HFrEF group compared to the stable HFrEF group.
Yu (2024) (40)	HF population, China	Observational study	116	HF (80, 60.9±9.62), HC (36, 54.8±8.88)	NT-proBNP	Diabetes mellitus (28.75%), hypertension (72.5%), atrial fibrillation (30%), chronic renal failure (13.75%), myocardial infarction	NT-proBNP levels are closely related to HF severity, NYHA grade, end-diastolic pressure, and the degree of hemodynamic disturbance.

		(17.5%), cerebral stroke (21.25%)

Supplementary Table 4. Studies assessing myocardial injury biomarkers for CHF

Study	Study population	Study design	N, subjects	Subject subgroups (N, Age)	Myocardial injury markers	Comorbidities	Conclusion of research
Aulin (2022) (18)	Atrial fibrillation with HF, Sweden	Randomized clinical trial	11818	HFrEF (2048, 67.0 (60.0 - 74.0)), HFpEF (2520, 69.0 (61.0 - 74.0)), HC (7250, 70.0 (63.0-76.0))	hs-cTnT, cystatin C	Diabetes mellitus (25%), hypertension (86.9%)	hs-cTnT and cystatin C levels were highest in the HFrEF group, followed by HFpEF, and lowest in healthy controls. hs-cTnT and cystatin C levels were independently associated with HF hospitalization and death.
Davarzani (2018) (8)	CHF population, Switzerland	Randomized controlled trial	499	No event CHF during last 19 months (312, 75.1±7.5), One or more events CHF (187, 77.9±7.2)	hs-cTnT, cystatin C	-	The event CHF cohort had a higher age, NYHA group, hs-cTnT and cystatin C levels compared to the no-event CHF group.
Gohar (2017) (73)	HF population, Singapore	Observational study	1096	HFrEF (853, 60.2±11.8), HFpEF (243, 68.3±11.4)	hs-TnT, hs-TnI	Coronary artery disease (52.5%), atrial fibrillation (22.5%), hypertension (71.9%), diabetes mellitus (57%)	Both TnT and TnI are elevated in HF and independently predict poorer outcomes. Their prognostic value is stronger in HFpEF than in HFrEF, with hs-TnT showing the best performance in HFpEF. Notably, hs-TnI is a better predictor in men than in women, highlighting a sex-specific aspect.

Akiyama (2020) (52)	HF population, France	Observational study	59	HFpEF (34, 83 (76–89)), HFrEF (25, 63 (57–76))	hs-TnI, cystatin C	-	No differences in hs-TnI and cystatin C levels between phenotype groups were observed.
Dubrock (2018) (27)	HF population, USA	Clinical trial	214	Normal hs-CRP HFpEF (93, 70 (65-79)), high hs-CRP HFpEF (121, 67 (61-76))	hs-TnI, cystatin C	Diabetes mellitus (43%), hypertension (85%), ischemic heart disease (38%), Atrial fibrillation (51%), COPD (20%), anemia (40%).	There is no association between hs-TnI and cystatin C with hs-CRP concentration.
Mohebi (2022a) (33)	HF population, USA	Observational study	1235	Stage A HF (77, 57.3±8.9), stage B HF (733, 66±11.3), stage C/D HF (425, 68.8±11.5)	hs-cTnI, cystatin C	Type 2 diabetes (25.58%), hypertension (75.46%), hyperlipidemia (66.8%), CKD (13%), COPD (17.73%), myocardial infarction (23.24%), atrial fibrillation (18.62%)	hs-cTnI and cystatin C levels significantly increase with HF severity. hs-cTnI level was not associated with HF or CV death in stage C/D HF.
Askevold (2013) (15)	CHF population, Norway	Randomized controlled trial	1455	CHF (1455, 71.8±6.9)	TnT	Hypertension (69%), diabetes mellitus (26%), atrial fibrillation (21.9%), myocardial infarction (63.1%)	Troponin T level increases with higher levels of sgp130.
Brankovic (2019) (66)	Chronic HF population, Netherlands	Clinical trial	263	Composite endpoint CHF (70, 69±13), no composite endpoint CHF (193, 66±12)	hs-cTnT	Atrial fibrillation (40%), myocardial infarction (36%), diabetes (31%), hypercholesterolemia (36%), hypertension (46%), COPD (12%)	hs-cTnT level is significantly higher in the composite endpoint CHF group compared to the no composite endpoint CHF group.
De Marco (2021) (23)	HF population, USA/Canada /Russia	Randomized clinical trial	248	HFpEF with diabetes mellitus (116, 69±9.3), HFpEF without	hs-TnT	Cardiovascular disease (55.24%), myocardial infarction (21.37%), hypertension	Troponin T concentration shows no significant difference between HF

				diabetes mellitus (132, 74.3±9.7)		(93.55%), stroke (7.66%), COPD (11.3%), asthma (12.5%), dyslipidemia (78.22%), atrial fibrillation (49.6%)	groups with and without diabetes.
Drum (2017) (69)	HF population, Singapore	Observational study	657	HFrEF (219, 64.7±12), HFpEF (219, 68.2±11.2), HC (219, 65±9)	hs-TnT	Hypertension (75.8%), diabetes mellitus (57.5%), atrial fibrillation (26%)	hs-TnT concentration is highest in the HFrEF group and significantly lower in the healthy control group.
Ehteshami-Afshar (2021) (41)	Chronic HF population, Novartis Pharmaceuticals	Randomized controlled trial	8399	HFrEF without COPD (7319, 63.3±11.6), HFrEF with COPD (1080, 67.4±9.5)	hs-TnT	Hypertension (70.72%), atrial fibrillation (36.8%), myocardial infarction (43.26%), diabetes mellitus (34.48%), stroke (8.6%)	hs-TnT concentration was significantly higher in the HFrEF with COPD group.
Florea (2016) (28)	HF population, USA	Randomized controlled trial	3519	HFrEF (3198, 62±11), HF with improved EF (321, 61±11)	hs-TnT	Diabetes mellitus (25%), hypertension (6.5%), atrial fibrillation (11%), COPD (11.8%)	The HFiEF group has a lower hs-TnT concentration compared to the HFrEF group.
Galindo-Fraga (2003) (58)	HF population, Mexico	Observational study	43	NYHA I (24, 57±16), NYHA II (15, 65±16), NYHA III (4, 48±22)	TnT	Diabetes mellitus (50%), chronic renal failure (14%)	There is no significant difference in troponin T levels across NYHA classes.
Jungbauer (2014) (43)	Chronic HF population, Germany	Clinical trial	233	CHF (149, 61.8±11.6), HC (84, 42.9±8.3)	hs-TnT	-	A multimarker panel consisting of NT-proBNP, hs-TnT, TIMP-1, GDF-15, and IBP-4 provided improved prognostic information compared to the combination of NT-proBNP and hs-TnT alone. hs-TnT levels were

							significantly higher in CHF patients compared to healthy controls.
Lupón (2013) (45)	Chronic HF population, Spain	Clinical trial	876	Alive HF (565, 66.1 (56.5-74.3)), Deceased HF (311, 75.6 (69.9-81.0))	hs-TnT	Hypertension (61.2%), diabetes mellitus (35.8%), chronic obstructive lung disease (16.9%), sleep apnoea (4.5%), atrial fibrillation (16.7%)	hs-TnT levels were significantly higher in the deceased HF group compared to the alive HF group. The combination of hs-cTnT and hs-ST2 identified more decedents during follow-up compared to the combination of NT-proBNP, hs-cTnT, and hs-ST2.
Menghoum (2024) (46)	HF population, Belgium	Observational study	164	HFpEF (139, 78±8), HC (25, 77±5)	hs-TnT	Atrial fibrillation (60%), coronary artery disease (33%), myocardial infarction (12%), COPD (9%), sleep apnoea (13%)	hs-TnT levels were significantly higher in HFpEF compared to HC. hs-TnT levels were associated with higher carbohydrate antigen 125 (CA125).
Mohebi (2022b) (64)	HF population, USA	Observational study	794	HF duration <12 months (178, 61.8±13.1), HF 12-24 months (80, 65.2±14.6), HF 24-60 months (185, 64.3±12.7), HF >60 months (351, 67.3±10.7)	hs-cTnT	Hypertension (88%), stroke (10.8%), myocardial infarction (41.4%), diabetes mellitus (45.5%), atrial fibrillation (33.75%)	There is no association between hs-cTnT level and HF duration.
Pandhi (2022) (20)	Chronic HF population, Europe (Netherlands)	Observational study	550	No congestion CHF (408, 66.6 ± 11.7), Severe congestion CHF (142, 69.6 ± 12.8)	TnT	Diabetes (30%), hypertension (64%), atrial fibrillation (42%), COPD (14.5), renal disease (30%)	Troponin T concentration is significantly elevated in patients with severe congestive CHF

							compared to those without congestion.
Roy (2020) (47)	HF population, Belgium	Clinical trial	174	HFpEF (143, 78±8), HC (31, 75±6)	hs-TnT	Atrial fibrillation (62%), chronic obstructive pulmonary disease (10%), sleep apnoea (13%)	hs-TnT is a predictor of the primary endpoint. Its level is significantly higher in HFpEF compared to HC.
Teramoto (2024) (36)	Chronic HF, Japan	Randomized controlled trial	942	CHF under 50 years (73, 44 (39-47)), CHF 50-59 years (158, 56 (54-58)), CHF 60-69 years (296, 65.5 (63-80)), CHF 70-79 years (415, 75 (72-77))	Troponin	Diabetes (47.7%), dyslipidaemia (52%), ischaemic heart disease (49.3%), cardiomyopathy (25.5%), atrial fibrillation (41.5%)	Troponin levels increase with age in the CHF cohort.
Wołowiec (2023) (38)	HF population, Poland	Observational study	120	Death HFrEF (44, 69.91±13.74), survived HFrEF (76, 55.53±12.8)	TnT	-	Troponin T level is significantly higher in the deceased HFrEF group compared to the surviving HFrEF group.
Wołowiec (2024) (39)	HF population, Poland	Observational study	122	Stable HFrEF (52, 51.58±9.16), HFrEF with exacerbation (70, 67.93±14.38)	TnT	Diabetes mellitus (40.9%), hypertension (53.3%), atrial fibrillation (49.1%)	Baseline Troponin T concentration was higher in the exacerbation HFrEF group compared to the stable HFrEF group.
Chiu (2024) (61)	HF population, USA	Randomized controlled trial	212	NT-proBNP/cGMP Q1 (53, 62 (55-67)), Q2 (53, 68 (63-76)), Q3 (53, 73 (67-80)), Q4 (53, 73 (65-79))	TnI	Hypertension (84.9%), diabetes (42.9%), atrial fibrillation (51.4%), myocardial infarction (28.3%), COPD (28.3%)	Patients with a higher NT-proBNP/cGMP ratio had higher troponin I levels. Troponin I was significantly associated with the NT-proBNP/cGMP ratio but not with NT-proBNP alone.

Do e (2013) (25)	Chronic HF population, Japan	Clinical trial	160	CHF (130, 58±1), HC (30, 54±2)	hs-cTnI	-	There is no significant difference between the healthy control group and the CHF group.
Gaggin (2016) (42)	Chronic HF population, USA	Randomized controlled trial	108	HFrEF with cachexia (20, -), HFrEF without cachexia (88, -)	hs-TnI	-	Patients who developed cachexia during follow-up had higher concentrations of hs-TnI compared to those with HF without cachexia.
Hajsadeghi (2019) (78)	Decompensated HF population, Iran	Observational study	97	HF with rehospitalization (16, 48±16), HF without rehospitalization (72, 44±17)	hs-cTnI	Diabetes (16%), hypertension (37%)	Hs-cTnI levels are significantly higher in rehospitalized patients and in those with diabetes.
Ren (2023) (35)	HF population, China	Observational study	964	Event HF (232, 68±14), no-event HF (732, 62±14.4)	cTnI	Hypertension (57.8%), diabetes mellitus (35%), hyperlipidemia (32.4%), chronic kidney disease (18.2%), myocardial infarction (16.2%), stroke (13.7%), atrial fibrillation (28.8%)	cTnI levels are significantly higher in the event HF group compared to the no-event HF group.
Tromp (2017) (1)	Chronic HF population, Netherlands	Case control study	460	HFrEF (364, 69.6±11.2), HFpEF (96, 74.5±10.0)	cTnI	Atrial fibrillation (45.4%), diabetes mellitus (29.3%), COPD (28.3%), hypertension (41.5%), anemia (27.8%), myocardial infarction (40.7%)	There was no significant difference in cTnI concentration between HFrEF and HFpEF groups.
Tymińska (2019) (37)	Population without HF, Poland	Observational study	104	Without HF at 1 year (54, 58(43-67.3)), With HF at 1 year (50, 64(57-70.8))	cTnI	Hypertension (64%), atrial fibrillation (10%), diabetes (30%), chronic kidney disease (22%), COPD (6%)	cTnI level was higher in the LVEF <50% group compared to the LVEF >50% group. Baseline cTnI level

							was significantly higher in patients who developed HF within one year.
AbouEzzeddine (2017) (63)	HF population, USA	Clinical trial	174	Low ST2 HF (58, 67 (61-74)), Mid ST2 HF (58, 71 (64-79)), High ST2 HF (58, 69 (63-77))	Cystatin C	Hypertension (83.9%), atrial fibrillation (48.85%), COPD (18.4%), diabetes mellitus (40.8%)	Cystatin C level increases significantly with increasing ST2 tertiles.
Gao (2019) (72)	Chronic HF population, China	Observational study	344	HFrEF (168, 57.25±14.92), HC (176, 55.11±13.44)	Cystatin C	Myocardial infarction (31%), diabetes mellitus (23.8%), hypertension (39.3%)	Cystatin C level is significantly higher in the HFrEF group compared to the HC group.
Ge (2023) (29)	HF population, China	Observational study	276	HF (128, 61 (52-68)), HC (148, 61 (50-66))	Cystatin C	Hypertension (50%), diabetes mellitus (35.9%), hypercholesterolemia (10.9%), atrial fibrillation (25.8%), COPD (2.3%), myocardial infarction (48.4%), anemia (3.9%)	Cystatin C level is significantly higher in the HF group compared to the HC group.
Yu (2024) (40)	HF population, China	Observational study	116	HF (80, 60.9±9.62), HC (36, 54.8±8.88)	Cystatin C	Diabetes mellitus (28.75%), hypertension (72.5%), atrial fibrillation (30%), chronic renal failure (13.75%), myocardial infarction (17.5%), cerebral stroke (21.25%)	Cystatin C level is significantly higher in the HF group compared to healthy controls.

1. Tromp J, Khan MAF, Klip IT, Meyer S, de Boer RA, Jaarsma T, et al. Biomarker profiles in heart failure patients with preserved and reduced ejection fraction. Journal of the American Heart Association. 2017;6(4).
2. Boulogne M, Sadoune M, Launay JM, Baudet M, Cohen-Solal A, Logeart D. Inflammation versus mechanical stretch biomarkers over time in acutely decompensated heart failure with reduced ejection fraction. Int J Cardiol. 2017;226:53-9.
3. Abernethy A, Raza S, Sun JL, Anstrom KJ, Tracy R, Steiner J, et al. Pro-Inflammatory Biomarkers in Stable Versus Acutely Decompensated Heart Failure With Preserved Ejection Fraction. J Am Heart Assoc. 2018;7(8).
4. Almasood A, Sheshgiri R, Joseph JM, Rao V, Kamali M, Tumiati L, et al. Human leukocyte antigen-G is upregulated in heart failure patients: A potential novel biomarker. Human Immunology. 2011;72(11):1064-7.
5. Pryds K, Rahbek Schmidt M, Bjerre M, Thiel S, Refsgaard J, Bøtker HE, et al. Effect of long-term remote ischemic conditioning on inflammation and cardiac remodeling. Scand Cardiovasc J. 2019;53(4):183-91.
6. Stanciu AE, Stanciu MM, Vatasescu RG. NT-proBNP and CA 125 levels are associated with increased pro-inflammatory cytokines in coronary sinus serum of patients with chronic heart failure. Cytokine. 2018;111:13-9.
7. Susa T, Kobayashi S, Tanaka T, Murakami W, Akashi S, Kunitsugu I, et al. Urinary 8-hydroxy-2'-deoxyguanosine as a novel biomarker for predicting cardiac events and evaluating the effectiveness of carvedilol treatment in patients with chronic systolic heart failure. Circulation Journal. 2012;76(1):117-26.
8. Davarzani N, Sanders-van Wijk S, Maeder MT, Rickenbacher P, Smirnov E, Karel J, et al. Novel concept to guide systolic heart failure medication by repeated biomarker testing—results from TIME-CHF in context of predictive, preventive, and personalized medicine. EPMA Journal. 2018;9(2):161-73.
9. Everett BM, Cornel JH, Lainscak M, Anker SD, Abbate A, Thuren T, et al. Anti-Inflammatory Therapy With Canakinumab for the Prevention of Hospitalization for Heart Failure. Circulation. 2019;139(10):1289-99.
10. Fedacko J, Singh RB, Gupta A, Hristova K, Toda E, Kumar A, et al. Inflammatory mediators in chronic heart failure in North India. Acta Cardiologica. 2014;69(4):391-8.
11. Nakamura T, Funayama H, Kubo N, Yasu T, Kawakami M, Momomura Si, et al. Elevation of plasma placental growth factor in the patients with ischemic cardiomyopathy. International Journal of Cardiology. 2009;131(2):186-91.
12. Niebauer J, Clark AL, Webb-Peploe KM, Coats AJ. Exercise training in chronic heart failure: effects on pro-inflammatory markers. Eur J Heart Fail. 2005;7(2):189-93.
13. Richter B, Koller L, Hohensinner PJ, Zorn G, Brekalo M, Berger R, et al. A multi-biomarker risk score improves prediction of long-term mortality in patients with advanced heart failure. International Journal of Cardiology. 2013;168(2):1251-7.
14. White M, Ducharme A, Ibrahim R, Whittom L, Lavoie J, Guertin MC, et al. Increased systemic inflammation and oxidative stress in patients with worsening congestive heart failure: improvement after short-term inotropic support. Clin Sci (Lond). 2006;110(4):483-9.
15. Askevold ET, Nymo S, Ueland T, Gravning J, Wergeland R, Kjekshus J, et al. Soluble glycoprotein 130 predicts fatal outcomes in chronic heart failure: analysis from the Controlled Rosuvastatin Multinational Trial in Heart Failure (CORONA). Circ Heart Fail. 2013;6(1):91-8.
16. Grossman GB, Rohde LE, Clausell N. Evidence for increased peripheral production of tumor necrosis factor-alpha in advanced congestive heart failure. Am J Cardiol. 2001;88(5):578-81.
17. Sobieszek G, Powrózek T, Mazurek M, Skwarek-Dziekanowska A, Małecka-Massalska T. Electrical and hormonal biomarkers in cachectic elderly women with chronic heart failure. Journal of Clinical Medicine. 2020;9(4).
18. Aulin J, Hijazi Z, Lindbäck J, Alexander JH, Gersh BJ, Granger CB, et al. Biomarkers and heart failure events in patients with atrial fibrillation in the ARISTOTLE trial evaluated by a multi-state model. Am Heart J. 2022;251:13-24.

19. Kaye DM, Nanayakkara S, Wang B, Shihata W, Marques FZ, Esler M, et al. Characterization of Cardiac Sympathetic Nervous System and Inflammatory Activation in HFpEF Patients. JACC: Basic to Translational Science. 2022;7(2):116-27.
20. Pandhi P, ter Maaten JM, Anker SD, Ng LL, Metra M, Samani NJ, et al. Pathophysiologic Processes and Novel Biomarkers Associated With Congestion in Heart Failure. JACC: Heart Failure. 2022;10(9):623-32.
21. Wang W, Zhang X, Ge N, Liu J, Yuan H, Zhang P, et al. Procalcitonin testing for diagnosis and short-term prognosis in bacterial infection complicated by congestive heart failure: A multicenter analysis of 4,698 cases. Critical Care. 2014;18(1).
22. Cakmak HA, Coskunpinar E, Ikitimur B, Barman HA, Karadag B, Tiryakioglu NO, et al. The prognostic value of circulating microRNAs in heart failure: Preliminary results from a genome-wide expression study. Journal of Cardiovascular Medicine. 2015;16(6):431-7.
23. De Marco C, Claggett BL, de Denus S, Zile MR, Huynh T, Desai AS, et al. Impact of diabetes on serum biomarkers in heart failure with preserved ejection fraction: insights from the TOPCAT trial. ESC Heart Failure. 2021;8(2):1130-8.
24. Djoussé L, Matsumoto C, Petrone A, Weir NL, Tsai MY, Gaziano JM. Plasma galectin 3 and heart failure risk in the Physicians' Health Study. Eur J Heart Fail. 2014;16(3):350-4.
25. Do.e Z, Fukumoto Y, Sugimura K, Miura Y, Tatebe S, Yamamoto S, et al. Rho-kinase activation in patients with heart failure. Circulation Journal. 2013;77(10):2542-50.
26. Dudek M, Kałużna-Oleksy M, Migaj J, Sawczak F, Krysztofiak H, Lesiak M, et al. sST2 and Heart Failure—Clinical Utility and Prognosis. Journal of Clinical Medicine. 2023;12(9).
27. DuBrock HM, AbouEzzeddine OF, Redfield MM. High-sensitivity C-reactive protein in heart failure with preserved ejection fraction. PLoS One. 2018;13(8):e0201836.
28. Florea VG, Rector TS, Anand IS, Cohn JN. Heart failure with improved ejection fraction: clinical characteristics, correlates of recovery, and survival. Circulation: Heart Failure. 2016;9(7).
29. Ge Y, Liu X, Chen H, Li G, Xing X, Liu J, et al. The serum soluble scavenger with 5 domains levels: A novel biomarker for individuals with heart failure. Frontiers in Physiology. 2023;14.
30. Gravning J, Askevold ET, Nymo SH, Ueland T, Wikstrand J, McMurray JJ, et al. Prognostic effect of high-sensitive troponin T assessment in elderly patients with chronic heart failure: results from the CORONA trial. Circ Heart Fail. 2014;7(1):96-103.
31. Loncar G, Bozic B, Cvorovic V, Radojicic Z, Dimkovic S, Markovic N, et al. Relationship between RANKL and neuroendocrine activation in elderly males with heart failure. Endocrine. 2010;37(1):148-56.
32. Loncar G, von Haehling S, Tahirovic E, Inkrot S, Mende M, Sekularac N, et al. Effect of beta blockade on natriuretic peptides and copeptin in elderly patients with heart failure and preserved or reduced ejection fraction: results from the CIBIS-ELD trial. Clin Biochem. 2012;45(1-2):117-22.
33. Mohebi R, Murphy S, Jackson L, McCarthy C, Abboud A, Murtagh G, et al. Biomarker prognostication across Universal Definition of Heart Failure stages. ESC Heart Failure. 2022;9(6):3876-87.
34. Rathcke CN, Kistorp C, Raymond I, Hildebrandt P, Gustafsson F, Lip GYH, et al. Plasma YKL-40 levels are elevated in patients with chronic heart failure. Scandinavian Cardiovascular Journal. 2010;44(2):92-9.
35. Ren L, Li F, Tan X, Fan Y, Ke B, Zhang Y, et al. Abnormal plasma ceramides refine high-risk patients with worsening heart failure. Frontiers in Cardiovascular Medicine. 2023;10.
36. Teramoto K, Nochioka K, Sakata Y, Nishimura K, Shimokawa H, Yasuda S. Prognostic significance of growth differentiation factor-15 across age in chronic heart failure. ESC Heart Failure. 2024;11(3):1666-76.
37. Tymińska A, Kapłon-Cieślicka A, Ozierański K, Budnik M, Wancerz A, Sypień P, et al. Association of Galectin-3 and Soluble ST2, and Their Changes, with Echocardiographic Parameters and Development of Heart Failure after ST-Segment Elevation Myocardial Infarction. Dis Markers. 2019;2019:9529053.
38. Wołowiec Ł, Banach J, Budzyński J, Wołowiec A, Kozakiewicz M, Bieliński M, et al. Prognostic Value of Plasma Catestatin Concentration in Patients with Heart Failure with Reduced Ejection Fraction in Two-Year Follow-Up. Journal of Clinical Medicine. 2023;12(13).

39. Wołowiec Ł, Rogowicz D, Budzyński J, Banach J, Wołowiec A, Kozakiewicz M, et al. Prognostic value of plasma secretoneurin concentration in patients with heart failure with reduced ejection fraction in one-year follow-up. Annals of Medicine. 2024;56(1).
40. Yu ZL, Cai ZH, Zheng JT, Jiang HY, Zhou YQ, Wong NK, et al. Serum fibroblast growth factor-2 levels complement vital biomarkers for diagnosing heart failure. BMC Cardiovascular Disorders. 2024;24(1).
41. Ehteshami-Afshar S, Mooney L, Dewan P, Desai AS, Lang NN, Lefkowitz MP, et al. Clinical Characteristics and Outcomes of Patients With Heart Failure With Reduced Ejection Fraction and Chronic Obstructive Pulmonary Disease: Insights From PARADIGM-HF. J Am Heart Assoc. 2021;10(4):e019238.
42. Gaggin HK, Belcher AM, Gandhi PU, Ibrahim NE, Januzzi JL, Jr. Serial Echocardiographic Characteristics, Novel Biomarkers and Cachexia Development in Patients with Stable Chronic Heart Failure. J Cardiovasc Transl Res. 9. United States2016. p. 429-31.
43. Jungbauer CG, Riedlinger J, Block D, Stadler S, Birner C, Buesing M, et al. Panel of emerging cardiac biomarkers contributes for prognosis rather than diagnosis in chronic heart failure. Biomark Med. 2014;8(6):777-89.
44. Ahmad F, Karim A, Khan J, Qaisar R. Circulating H-FABP as a biomarker of frailty in patients with chronic heart failure. Experimental Biology and Medicine. 2023;248(16):1383-92.
45. Lupón J, de Antonio M, Galán A, Vila J, Zamora E, Urrutia A, et al. Combined use of the novel biomarkers high-sensitivity troponin T and ST2 for heart failure risk stratification vs conventional assessment. Mayo Clin Proc. 2013;88(3):234-43.
46. Menghoum N, Badii MC, Deltombe M, Lejeune S, Roy C, Vancraeynest D, et al. Carbohydrate antigen 125: a useful marker of congestion, fibrosis, and prognosis in heart failure with preserved ejection fraction. ESC Heart Failure. 2024;11(3):1493-505.
47. Roy C, Lejeune S, Slimani A, de Meester C, Ahn As SA, Rousseau MF, et al. Fibroblast growth factor 23: a biomarker of fibrosis and prognosis in heart failure with preserved ejection fraction. ESC Heart Failure. 2020;7(5):2494-507.
48. Mendez Fernandez AB, Ferrero-Gregori A, Garcia-Osuna A, Mirabet-Perez S, Pirla-Buxo MJ, Cinca-Cuscullola J, et al. Growth differentiation factor 15 as mortality predictor in heart failure patients with non-reduced ejection fraction. ESC Heart Failure. 2020;7(5):2223-9.
49. Claus R, Berliner D, Bavendiek U, Vodovar N, Lichtinghagen R, David S, et al. Soluble neprilysin, NT-proBNP, and growth differentiation factor-15 as biomarkers for heart failure in dialysis patients (SONGBIRD). Clinical Research in Cardiology. 2020;109(8):1035-47.
50. Poglajen G, Ksela J, Frljak S, Zemljic G, Boznar Alic E, Cerar A, et al. Favorable Response to CD34+ Cell Therapy Is Associated with a Decrease of Galectin-3 Levels in Patients with Chronic Heart Failure. Dis Markers. 2019;2019:8636930.
51. Wright SP, Doughty RN, Frampton CM, Gamble GD, Yandle TG, Richards AM. Plasma urocortin 1 in human heart failure. Circ Heart Fail. 2009;2(5):465-71.
52. Akiyama E, Cinotti R, Čerlinskaitė K, Van Aelst LNL, Arrigo M, Placido R, et al. Improved cardiac and venous pressures during hospital stay in patients with acute heart failure: an echocardiography and biomarkers study. ESC Heart Failure. 2020;7(3):996-1006.
53. Bahuleyan CG, Alummoottil GK, Abdullakutty J, Lordson AJ, Babu S, Krishnakumar VV, et al. Prognostic value of soluble ST2 biomarker in heart failure patients with reduced ejection fraction – A multicenter study. Indian Heart Journal. 2018;70:S79-S84.
54. Crnko S, Printezi MI, Jansen TPJ, Leiteris L, van der Meer MG, Schutte H, et al. Prognostic biomarker soluble ST2 exhibits diurnal variation in chronic heart failure patients. ESC Heart Failure. 2020;7(3):1224-33.
55. Firouzabadi N, Dashti M, Dehshahri A, Bahramali E. Biomarkers of IL-33 and SST2 and lack of association with carvedilol therapy in heart failure. Clinical Pharmacology: Advances and Applications. 2020;12:53-8.
56. Gruson D, Lepoutre T, Ahn SA, Rousseau MF. Increased soluble ST2 is a stronger predictor of long-term cardiovascular death than natriuretic peptides in heart failure patients with reduced ejection fraction. International Journal of Cardiology. 2014;172(1):e250-e2.

57. Andersson SE, Edvinsson ML, Alving K, Edvinsson L. Vasodilator effect of endothelin in cutaneous microcirculation of heart failure patients. Basic Clin Pharmacol Toxicol. 2005;97(2):80-5.
58. Galindo-Fraga A, Arrieta O, Castillo-Martínez L, Narváez R, Oseguera-Moguel J, Orea-Tejeda A. Elevation of plasmatic endothelin in patients with heart failure. Archives of Medical Research. 2003;34(5):367-72.
59. Behnes M, Brueckmann M, Lang S, Espeter F, Weiss C, Neumaier M, et al. Diagnostic and prognostic value of osteopontin in patients with acute congestive heart failure. European Journal of Heart Failure. 2013;15(12):1390-400.
60. Coculescu BI, Manole G, Dincă GV, Coculescu EC, Berteanu C, Stocheci CM. Osteopontin–a biomarker of disease, but also of stage stratification of the functional myocardial contractile deficit by chronic ischaemic heart disease. Journal of Enzyme Inhibition and Medicinal Chemistry. 2019;34(1):783-8.
61. Chiu L, Agrawal V, Armstrong D, Brittain E, Collins S, Hemnes AR, et al. Correlates of Plasma NT-proBNP/Cyclic GMP Ratio in Heart Failure With Preserved Ejection Fraction: An Analysis of the RELAX Trial. J Am Heart Assoc. 2024;13(7):e031796.
62. Gocer H, Günday M, Ünal M. Plasma galectin-3 as a biomarker for clinical staging of heart failure: A cross-sectional evaluation of 100 cases. Clinica Terapeutica. 2019;170(4):e267-e71.
63. AbouEzzeddine OF, McKie PM, Dunlay SM, Stevens SR, Felker GM, Borlaug BA, et al. Suppression of tumorigenicity 2 in heart failure with preserved ejection fraction. Journal of the American Heart Association. 2017;6(2).
64. Mohebi REZA, Liu YUXI, Felker GM, Prescott MF, Ward JH, PiÑA IL, et al. Heart Failure Duration and Mechanistic Efficacy of Sacubitril/Valsartan in Heart Failure With Reduced Ejection Fraction. Journal of Cardiac Failure. 2022;28(12):1673-82.
65. Bai Y, Zhang P, Zhang X, Huang J, Hu S, Wei Y. LTBP-2 acts as a novel marker in human heart failure a preliminary study. Biomarkers. 2012;17(5):407-15.
66. Brankovic M, Martijn Akkerhuis K, Mouthaan H, Constantinescu A, Caliskan K, van Ramshorst J, et al. Utility of temporal profiles of new cardio-renal and pulmonary candidate biomarkers in chronic heart failure. Int J Cardiol. 2019;276:157-65.
67. Chenevier-Gobeaux C, Delerme S, Allo JC, Arthaud M, Claessens YE, Ekindjian OG, et al. B-type natriuretic peptides for the diagnosis of congestive heart failure in dyspneic oldest-old patients. Clinical Biochemistry. 2008;41(13):1049-54.
68. Cristóbal H, Enjuanes C, Batlle M, Tajes M, Campos B, Francesch J, et al. Prognostic Value of Soluble AXL in Serum from Heart Failure Patients with Preserved and Reduced Left Ventricular Ejection Fraction. Journal of Personalized Medicine. 2023;13(3).
69. Drum CL, Tan WKY, Chan SP, Pakkiri LS, Chong JPC, Liew OW, et al. Thymosin beta-4 is elevated in women with heart failure with preserved ejection fraction. Journal of the American Heart Association. 2017;6(6).
70. Ekure EN, Okoromah CA, Ajuluchukwu JN, Mbakwem A, Oladipo J. Diagnostic usefulness of N-terminal pro-brain natriuretic peptide among children with heart failure in a Tertiary hospital in Lagos, Nigeria. West African Journal of Medicine. 2011;30(1):30-4.
71. Fröhling T, Semo D, Mirna M, Paar V, Shomanova Z, Motloch LJ, et al. Novel Biomarkers as Potential Predictors of Decompensated Advanced Chronic Heart Failure—Single Center Study. Journal of Clinical Medicine. 2024;13(22).
72. Gao C, Zhao S, Lian K, Mi B, Si R, Tan Z, et al. C1q/TNF-related protein 3 (CTRP3) and 9 (CTRP9) concentrations are decreased in patients with heart failure and are associated with increased morbidity and mortality. BMC Cardiovascular Disorders. 2019;19(1).
73. Gohar A, Chong JPC, Liew OW, den Ruijter H, de Kleijn DPV, Sim D, et al. The prognostic value of highly sensitive cardiac troponin assays for adverse events in men and women with stable heart failure and a preserved vs. reduced ejection fraction. European Journal of Heart Failure. 2017;19(12):1638-47.
74. Maeder MT, Rickenbacher P, Rickli H, Abbühl H, Gutmann M, Erne P, et al. N-terminal pro brain natriuretic peptide-guided management in patients with heart failure and preserved ejection fraction: Findings from the Trial of Intensified versus standard Medical therapy in Elderly patients with Congestive Heart Failure (TIME-CHF). European Journal of Heart Failure. 2013;15(10):1148-56.

75. Obokata M, Kane GC, Reddy YNV, Melenovsky V, Olson TP, Jarolim P, et al. The neurohormonal basis of pulmonary hypertension in heart failure with preserved ejection fraction. European Heart Journal. 2019;40(45):3707-17.
76. Thibodeau JT, Pham DD, Kelly SA, Ayers CR, Garg S, Grodin JL, et al. Subclinical Myocardial Injury and the Phenotype of Clinical Congestion in Patients With Heart Failure and Reduced Left Ventricular Ejection Fraction. Journal of Cardiac Failure. 2022;28(3):422-30.
77. van Wezenbeek J, Canada JM, Ravindra K, Carbone S, Trankle CR, Kadariya D, et al. C-Reactive Protein and N-Terminal Pro-brain Natriuretic Peptide Levels Correlate With Impaired Cardiorespiratory Fitness in Patients With Heart Failure Across a Wide Range of Ejection Fraction. Frontiers in Cardiovascular Medicine. 2018;5.
78. Hajsadeghi S, Bagheri Y, Ghafouri MH, Kerman SRJ, Hassanzadeh M. High-sensitive troponin I and re-hospitalization in patients with decompensated congestive heart failure. Acta Medica Iranica. 2019;57(2):116-21.
