# Supplementary figures and images for: Diagnostic and prognostic value of circulating biomarkers in heart failure
Source: Front Cardiovasc Med. 2025 Sep 24;12:1633164. doi: 10.3389/fcvm.2025.1633164 (PMC12504382; doi:10.3389/fcvm.2025.1633164)

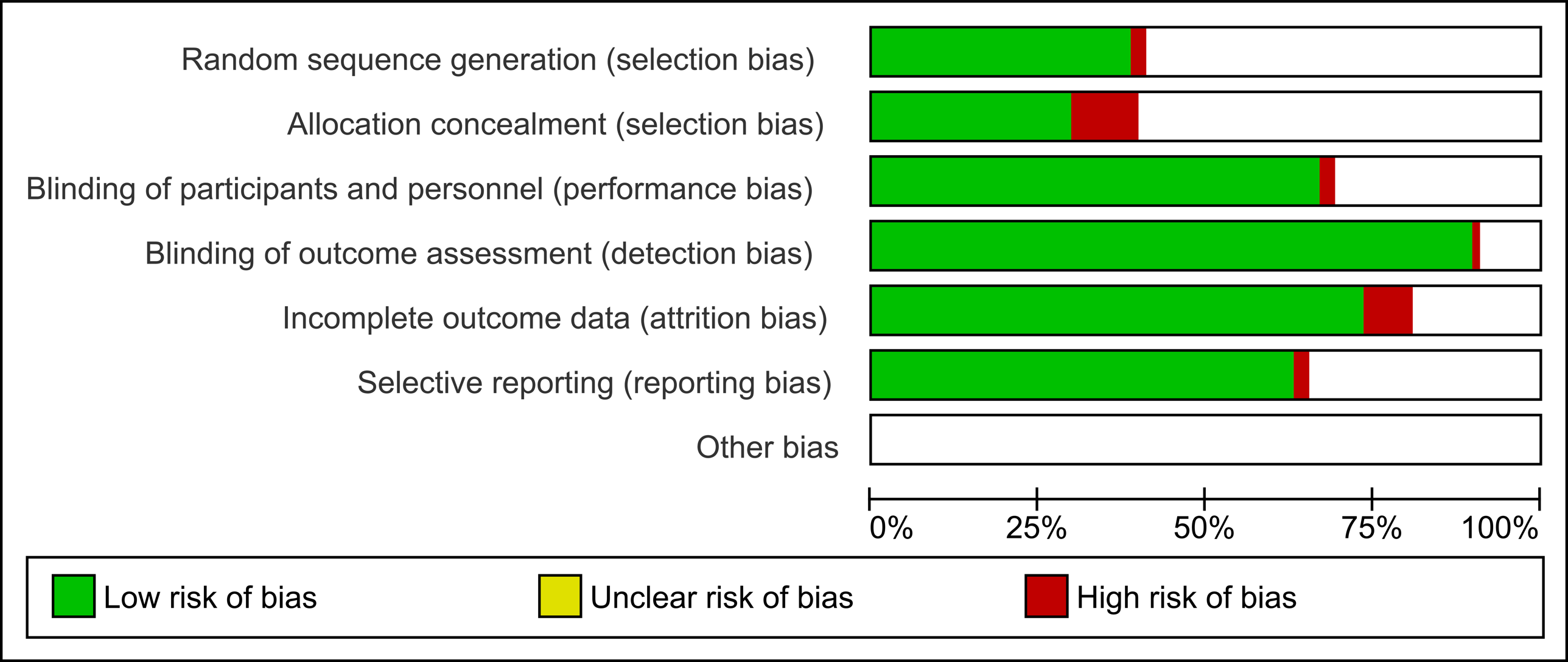

Supplement: Supplementary Figure S1 — Risk of bias graph. [file Image1.tiff]
